# Assessment of assumptions underlying models of prokaryotic pangenome evolution

**DOI:** 10.1186/s12915-021-00960-2

**Published:** 2021-02-10

**Authors:** Itamar Sela, Yuri I. Wolf, Eugene V. Koonin

**Affiliations:** National Center for Biotechnology Information, National Library of Medicine, National Institutes of Health, Bethesda, MD 20894 USA

**Keywords:** Evolutionary genomics, Bacterial evolution, Pangenome, Quantitative biology

## Abstract

**Background:**

The genomes of bacteria and archaea evolve by extensive loss and gain of genes which, for any group of related prokaryotic genomes, result in the formation of a pangenome with the universal, asymmetrical U-shaped distribution of gene commonality. However, the evolutionary factors that define the specific shape of this distribution are not thoroughly understood.

**Results:**

We investigate the fit of simple models of genome evolution to the empirically observed gene commonality distributions and genome intersections for 33 groups of closely related bacterial genomes. A model with an infinite external gene pool available for gene acquisition and constant genome size (IGP-CGS model), and two gene turnover rates, one for slow- and the other one for fast-evolving genes, allows two approaches to estimate the parameters for gene content dynamics. One is by fitting the model prediction to the distribution of the number of genes shared by precisely *k* genomes (gene commonality distribution) and another by analyzing the distribution of the number of genes common for *k* genome sets (*k*-cores). Both approaches produce a comparable overall quality of fit, although the former significantly overestimates the number of the universally conserved genes, while the latter overestimates the number of singletons. We further explore the effect of dropping each of the assumptions of the IGP-CGS model on the fit to the gene commonality distributions and show that models with either a finite gene pool or unequal rates of gene loss and gain (greater gene loss rate) eliminate the overestimate of the number of singletons or the core genome size.

**Conclusions:**

We examine the assumptions that are usually adopted for modeling the evolution of the U-shaped gene commonality distributions in prokaryote genomes, namely, those of infinitely many genes and constant genome size. The combined analysis of genome intersections and gene commonality suggests that at least one of these assumptions is invalid. The violation of both these assumptions reflects the limited ability of prokaryotes to gain new genes. This limitation seems to stem, at least partly, from the horizontal gene transfer barrier, i.e., the cost of accommodation of foreign genes by prokaryotes. Further development of models taking into account the complexity of microbial evolution is necessary for an improved understanding of the evolution of prokaryotes.

**Supplementary Information:**

The online version contains supplementary material available at 10.1186/s12915-021-00960-2.

## Background

With the accumulation of complete prokaryotic genomes, it has become evident that even closely related prokaryotes can substantially differ in their gene repertoires [1, 2]. Accordingly, for a collection of genomes, often, collectively construed as a species, it is natural to consider the pangenome, which is defined as the entire non-redundant gene repertoire spanned by the constituent genomes [3]. Reconstruction of the evolutionary dynamics of microbial pangenomes is essential for understanding the evolution of the traits of microbes including ecology, pathogenicity, and resistance.

**Fig. 1:**
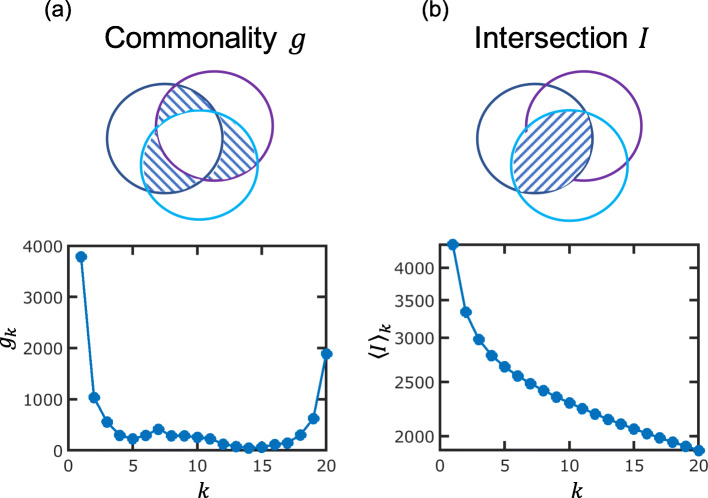
Gene commonality and genome intersection. The Venn diagrams illustrate the overlap of gene contents between genomes. The shaded areas demonstrate the difference between gene commonality (**a**) and genome intersection (**b**). Note that whereas *g*_2_ (intersection between two genomes) is a single number, for three genomes, there are three pairwise intersections, and only one of the three possibilities is indicated by the shaded area. In **a**, the common gene core of the three genomes is shown by the white area in the middle

The pangenome consists of genes of widely different abundances. Roughly, the genes in a pangenome can be divided into three classes according to their abundance: (1) the core, which is the collection of genes that are present in (nearly) all genomes; (2) the moderately conserved “shell”; and (3) the “cloud” of rare and unique genes [4]. To analyze gene abundances and their evolution quantitatively, it is convenient to analyze the distribution of gene commonality (this quantity is often referred to as gene frequency [5–9]; however, we prefer the term “commonality” to emphasize gene sharing among genomes). Gene commonality, *g*_*k*_, is defined for a collection of *N* genomes as the number of genes that are present in exactly *k* genomes, where *k* = 1, 2, …, *N*. The distribution of gene commonality is typically U-shaped, with different degrees of asymmetry (Fig. 1a), where the right peak corresponds to the conserved core, the shallow region in the middle to the moderately conserved shell, and the left peak to the cloud of rare genes [4]. In this representation, the pangenome is given by the sum of all points.

Prokaryotic genome evolution involves extensive gene loss and horizontal gene transfer (HGT) [10–14], which result in the formation of the characteristic structures of the pangenome and the core genome [15, 16]. Accordingly, the evolutionary models that account for the shaping of the gene commonality U-shaped distribution incorporate gene gain and loss rates. In a pioneering work of Baumdicker et al. [5], gene commonality was calculated under the assumption that genomes evolve around the equilibrium point where gain and loss rates are equal, such that the genome size is roughly constant. Inspired by the infinitely many alleles model of Kimura and Crow [17], the gene abundance was inferred under the assumption of the infinitely many genes (IMG) model. Under IMG, it is assumed that genes are acquired exclusively from an external, infinite gene pool, and every gene acquisition event introduces a new gene into the genetic repertoire. The IMG assumption was further implemented in different evolutionary models to study the formation of the U-shaped distribution in clusters of closely related bacterial genomes. It has been shown that at least two turnover rates of genes are required to accurately account for both the size of the core genome and the number of singleton genes [7]. Gene commonality was also studied in a general context of shared components in complex systems [8]. By considering complex systems of widely different nature and origins, including bacterial genomes, language texts, and Lego kits, it has been shown that the distribution of shared components has universal characteristics. In this work, genomes were regarded as random samples of genes, and the exact phyletic relations of the genomes were not incorporated into the analysis. However, more recently, it has been demonstrated that phyletic patterns could be inferred from the gene commonality distribution [18].

In previous studies [5–7], model parameters, in particular, gene turnover rates, were inferred from the gene commonality distribution. This approach requires explicit assumptions regarding the gene gain and loss rates. For example, the IMG model assumes that the gain rate is constant whereas the loss rate is proportional to the genome size [5, 6]. More importantly, fitting the model directly to gene commonality distribution can result in the inaccurate inference of turnover rates. Specifically, as we show in this work, the inference of the turnover rates from the gene commonality distribution will result in underestimation of the turnover rates due to the breakdown of the model assumptions.

Alternatively, gene turnover rates can be extracted directly from genome intersection *I*_*k*_, which is defined as the number of genes that are common to (at least) *k* genomes. Genome intersection is thus distinct from gene commonality *g*_*k*_, which is given by the number of genes that are present in exactly *k* genomes (Fig. 1). We have recently shown that genome intersection decays exponentially with the evolutionary distance [19, 20]. These findings are consistent with multiple pairwise comparisons of genomes in archaea [21], bacteria [22], and bacteriophages [23].

Here, we analyze the divergence of prokaryotic genomes within the theoretical framework we developed previously [19, 24] and extract gene commonality from genome intersection using the inclusion-exclusion principle [25]. This allows us to obtain the observed U-shaped distribution without assuming any specific functional form for the gain and loss rates. However, similar to the IMG model, it is assumed that gain and loss rates are equal and that genes are gained from an external infinite gene pool. Analysis of gene commonality and genome intersections in 33 groups of closely related prokaryotes indicates a greater number of gene losses compared to gene gain rates. This observation implies the breakdown of one or both of the model assumptions: either the actual gene gain rate is smaller than the loss rate, such that genomes shrink with time, or genes that are already present in the pangenome are often regained from the external pool that, in such a case, cannot be considered effectively infinite. Both these deviations from the model assumptions appear to be manifestations of the HGT barrier [26], that is, the cost of integrating new genes into the functional networks that already exist in the recipient organism.

## Results

### Extraction of gene commonality from genome intersections

Prokaryotic genome evolution is dominated by gene loss and HGT [10–14]. It is therefore natural to model genome evolution as a stochastic process where genes are gained and lost at random, with rates *P*^+^ and *P*^−^, respectively [24]. To understand better the role of selection in the model, it is convenient to further assume the weak mutation limit, where acquisition and deletion events are rare and appear sequentially. In this limit, the gain and loss rates can be written as [20, 24]:

1 $$ {P}^{+}=\alpha \cdot F(S) $$

2 $$ {P}^{-}=\beta \cdot F\left(-S\right) $$

where *α* and *β* are the acquisition and deletion rates, respectively, *F* is the fixation probability, and *S* is the selective benefit which is associated with the acquisition of one gene. The expressions of Eqs. 1 and 2 can be interpreted as the product of the contributions from mutation, selection, and drift. The mutations in this context are either acquisition or deletion of a gene, and selection is quantified by the fixation probability *F*. It should be stressed that the analysis of genome intersections and gene commonality presented below does not require the assumption of the weak mutation limit, but to make the role of selection in the model transparent, it is convenient to discuss it under this assumption.

Within this modeling framework, the intersection of *k* genomes, *I*_*k*_, decays exponentially with the total evolutionary distance *D*_*k*_ (see the “Methods” section for details). The exponential decay constant, *λ*, is given by the per-gene loss rate *λ*~*P*^−^/*x*, where *x* is the number of genes [19]. The exponential decay of genome intersections is obtained under two assumptions. First, it is assumed that gain and loss rates are closely similar, such that the number of genes is roughly constant. Second, it is assumed that genes are acquired from an infinite gene pool, such that each gain expands the pangenome by one new gene (hereafter infinite gene pool and constant genome size, or IGP-CGS assumptions).

Next, we have to take into account the evolutionary relationships among the intersecting genomes in a given cluster and calculate the mean intersection ⟨*I*⟩_*k*_ for each *k*. Evidently, in a cluster of *N* genomes, a subset of *k* genomes can be chosen in multiple ways (see the “Methods” section). The evolutionary relationships among the genomes are described by a phylogenetic tree, and for the calculation of ⟨*I*⟩_*k*_, the mean evolutionary distance for each *k* is weighted according to the tree topology (see the “Methods” section for explicit formulation). Using the inclusion-exclusion principle [25], it is possible to extract the gene commonality *g*_*k*_ from the mean genome intersection ⟨*I*⟩_*k*_:

3 $$ {g}_k={\sum}_{n=k}^N{\left(-1\right)}^{n-k}\bullet {q}_n^{(k)}\bullet {\left\langle I\right\rangle}_n $$

where

$$ {q}_n^{(k)}=\left(\begin{array}{c}n\\ {}k\end{array}\right)\bullet \left(\begin{array}{c}N\\ {}n\end{array}\right) $$

It should be noted that this relation is exact and does not rely on any approximation or assumption involved in the derivation of *I*_*k*_. This relation is not only a formal result but also provides an intuition with respect to the formation of the U-shaped distribution.

**Fig. 2:**
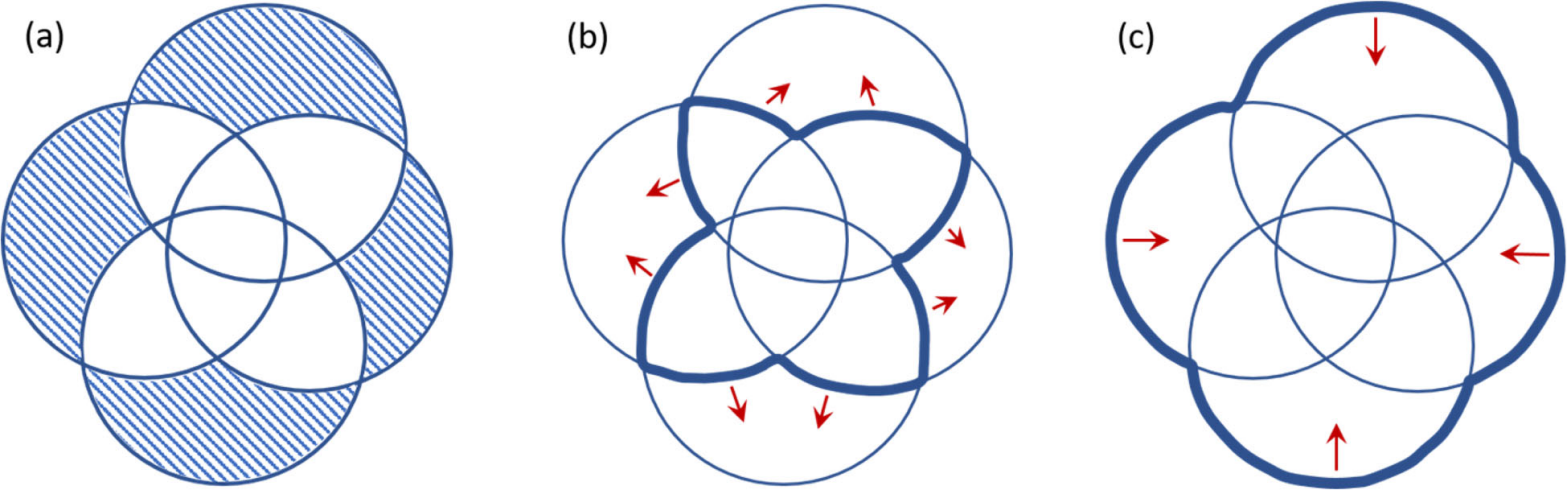
Venn diagrams illustrating how violation of either one of the IGP-CGS assumptions will reduce the number of singletons. The number of singletons can be extracted from gene intersections using Eq. 3 (**a**). When the assumption of an infinite gene pool is violated, genes can be regained into the intersection regions of the diagram, resulting in less singletons (**b**). Within the IGP-CGS model, the genome size is maintained by a balance of gene gain and gene loss events. When there are more gene losses than gene gains, the genome size reduces, decreasing the number of singletons (**c**)

Moreover, the combined analysis of genome intersections and gene commonality, and in particular, the relation encapsulated in Eq. 3, carries signatures of the evolutionary dynamics. For the core genome *k* = *N*, the sum of Eq. 3 contains only one term *g*_*N*_ = ⟨*I*⟩_*N*_. The core is given by the intersection that is associated with the longest evolutionary time, and its size determines the number of gene losses. For *k* < *N*, gene commonality is calculated backwards from the core genome, in an alternating sign series of *N* − *k* + 1 terms (see Eq. 3). The number of singletons *g*_1_ is given by a sum of *N* terms, with all intersections for *k* > 1 subtracted or added to the term *N* ∙ ⟨*I*⟩_1_, where ⟨*I*⟩_1_ is the mean genome size. The number of singletons is therefore sensitive to the genome size which, in the course of evolution, is maintained by gene gain. Under the CGS assumption, the number of gain events is equal to the number of loss events. Further assuming an infinite gene pool implies that all gained genes are initially singletons and contribute to *g*_1_. This means that the size of ⟨*I*⟩_1_ remains constant whereas all other ⟨*I*⟩_*k*_ with *k* > 1 are shrinking due to gene loss. As we show in the following section, extraction of gene commonality from genome intersections using the IGP-CGS model results in an overestimation of the number of singletons although the IGP-CGS model yields an excellent fit to the mean genome intersections (*R*^2^ > 0.996). This discrepancy implies violation of the IGP-CGS assumptions, as illustrated in Fig. 2. When the IGP assumption does not hold, genes can be regained from the finite external pool resulting in an increase of ⟨*I*⟩_*k*_ with *k* > 1 and a smaller number of singletons, as illustrated in Fig. 2b. Violation of the CGS assumption results in a decrease of the genome size, i.e., a lower ⟨*I*⟩_1_ value, and a smaller number of singletons as well (see Fig. 2c), even for the large number of gene losses that is implied by the size of the core genome.

### Analysis of simulated genome datasets

**Fig. 3:**
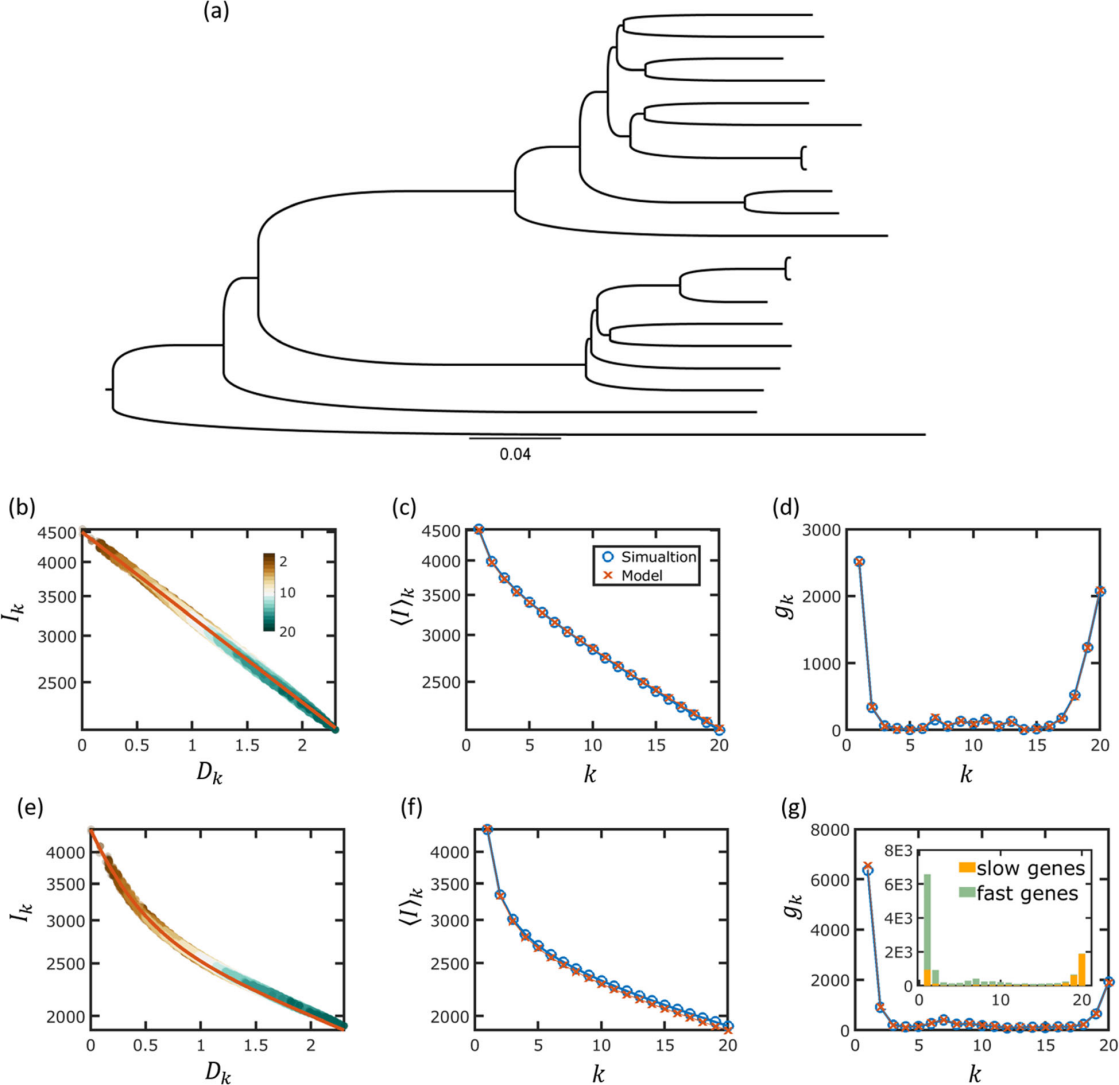
Estimation of genome intersection and gene commonality for a simulated genome dataset. **a** The phylogenetic tree used for generating the simulated dataset. **b** Genome intersections for a single turnover rate. Numbers of intersecting genomes are shown in different colors, as indicated by the color bar. The parameters used for the simulations are *x* = 4500 and *P*^+^ = *P*^−^ = 1.5 × 10^3^. The model prediction is obtained by substituting *x* and *P*^−^ into Eq. 7. Model prediction is indicated by a red line. **c** The mean genome intersections are calculated for the tree using Eq. 9. The simulated dataset is compared to model prediction (see legend). **d** Gene commonality for the simulated dataset and model prediction. The model prediction for gene commonality is extracted from the mean genome intersections of **b**, using Eq. 3. **e**–**g** Simulations using two turnover rates. Model parameters are identical to the values that were inferred for *E. coli* (see Additional file 1: Table S1). Panel content is analog to **b**–**d**. Model prediction for *I*_*k*_ was obtained using Eq. 8

To demonstrate the calculation stages, we perform the analysis for a simulated dataset (Fig. 3). Specifically, we aim to demonstrate the stages of gene commonality extraction from genome intersections. To simulate the evolution of genome content, we represent a genome as a collection of genes. Starting from the root and propagating along the tree branches, genes are lost and gained stochastically, according to the set gain and loss rates (for the complete description of the simulation scheme, see the “Methods” section). The simulations were performed for 20 genomes using the tree of the *Escherichia coli* cluster (Fig. 3a) and under the IGP-CGS assumptions.

Figure 3b–d shows the results of a single simulation using one gene turnover rate. The genome intersections and gene commonality values are extracted directly from the simulated dataset. For comparison, the analytical results are shown as well. In Fig. 3b, intersections are shown as a function of the evolutionary distance, as computed from the tree that was used for the simulation. For a single gene turnover rate, the intersections decay exponentially and therefore appear as straight lines in the semi-log plot of Fig. 3b. Next, the mean intersections ⟨*I*⟩_*k*_ are shown for all *k*’s in Fig. 3c. For the simulated dataset, ⟨*I*⟩_*k*_ is calculated for each *k* by taking the mean of all intersections *I*_*k*_. The model prediction for 〈*I*〉_*k*_ is obtained by averaging the exponential decay expression for *I*_*k*_, with weights that correspond to the tree topology (see the “Methods” section). Finally, the distribution of gene commonality *g*_*k*_ is shown in Fig. 3d, where model prediction for *g*_*k*_ is extracted from the mean genome intersections 〈*I*〉_*k*_ using Eq. 3.

To further explore the generality and validity of our modeling scheme, we repeated the entire procedure for a simulation with two gene turnover rates (Figs. 3e–g). As shown in the next section, two turnover rates were required to fit the model prediction for genome intersections, corresponding to two classes of genes, fast-evolving and slow-evolving ones. We therefore simulated the evolution of a genome that contains two classes of genes. In this case, the intersections (Fig. 3e) are given by a sum of two exponents (see the “Methods” section). The simulation was performed using realistic model parameters that were inferred from the analysis of the *E. coli* genome cluster (see Additional file 1: Table S1).

#### Selection in the model

Although selection is not explicitly incorporated in the model, this is not equal to the assumption that the genome evolution is neutral. Specifically, a model with a single class of genes does not assume neutral evolution. A single class of genes only implies that all genes are exchanged at the same rate, and accordingly, the selection coefficient *S* that quantifies the benefit of a single gene acquisition is the same for all genes. The interpretation of the pangenome evolutionary trajectory, in this case, is as follows. Initially, at the root of the tree, all genomes are identical, and therefore, all genes belong to the core. In the course of evolution, any gene loss event reduces the size of the core. In other words, due to the loss events, genes move from *k* = *n* to *k* < *n* occurrence, such that the core can be regarded as a source that diffuses to the left through gene loss. For short enough evolutionary times, a substantial fraction of the original core genome is retained, forming the observed right peak in Fig. 3d. On average, every time a gene is lost, a gene is gained. Due to the infinite gene pool assumption, each time a gene is gained, it is initially a singleton and contributes to *k* = 1 abundance. The gained genes form the left peak that consists of singletons and can be regarded as a source that diffuses from *k* = *n* to *k* > *n* occurrence through strain divergence and speciation.

As shown in the following, to fit the genomic data, two classes of genes are required. The different classes evolve under different turnover rates, reflecting different selective effects of acquisition or deletion of a gene between the two classes. In technical terms, the deletion rate *β* of Eq. 2 is the same for all genes and therefore for both classes, so that the different loss rates between the two gene classes are due to the difference in selective effects. Specifically, fast-evolving genes are associated with smaller selection coefficient compared to slow-evolving genes and are exchanged more easily [20]. As shown in Fig. 3e–g, the U-shaped core-shell-cloud structure of the gene commonality distribution is obtained, where fast and slow genes occupy different portions of the core, shell, and cloud regions, as shown in the inset of Fig. 3g.

### Fitting the model to the genomic data

After establishing the analysis strategy using simulated datasets, we applied the same calculation scheme to the genomic data. Specifically, we inferred gene turnover rates from the mean intersections ‹*I*›_*k*_ and compared the model predictions with the empirical gene commonality distributions. The analysis was performed with an extensive dataset that consisted of 33 clusters of closely related prokaryotic genomes (see the “Methods” section).

**Fig. 4:**
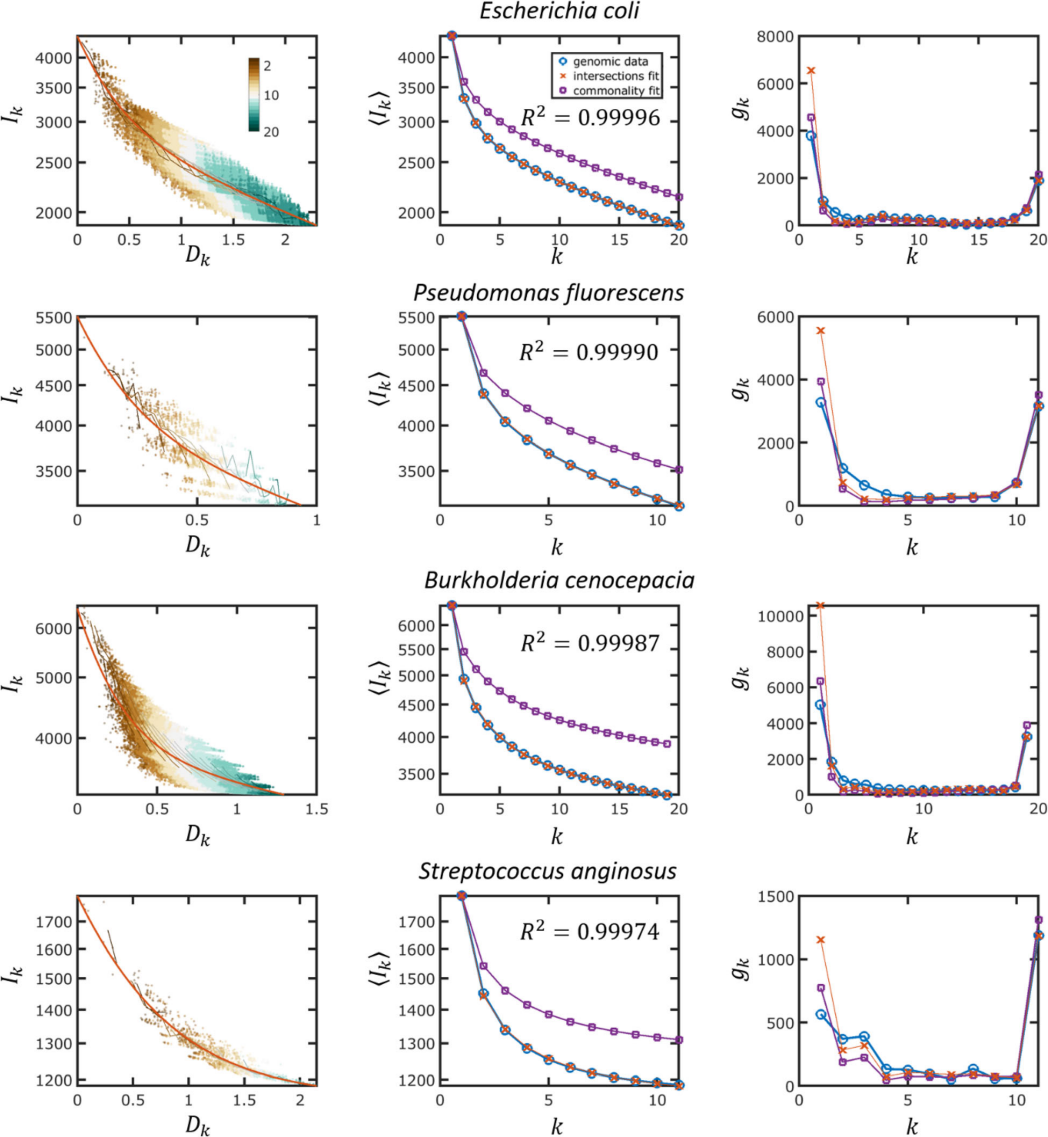
Empirical data and model fits of genome intersection and gene commonality for four genome clusters. The most common species in the cluster is indicated for each cluster. The panels on the left show the genome intersections, the panels in the middle show the mean genome intersections, and the panels on the right show the gene commonality (U-shaped distributions). The genomic data are shown by blue circles, and the model fits are shown by red x’s (genome intersection fit) or purple squares (gene commonality fit). The goodness of fit (*R*^2^) is indicated

In accord with the notion of core and accessory genes [16, 27], analysis of the genomic data shows that at least two turnover rates are required to fit the data (Fig. 4 and Additional file 1: Figs. S1-S6), indicating that genomes can be roughly divided into slow- (core) and fast- (accessory) evolving genes. Fitting the genomic data to a model with three classes of genes did not yield a better fit, as shown below.

**Fig. 5:**
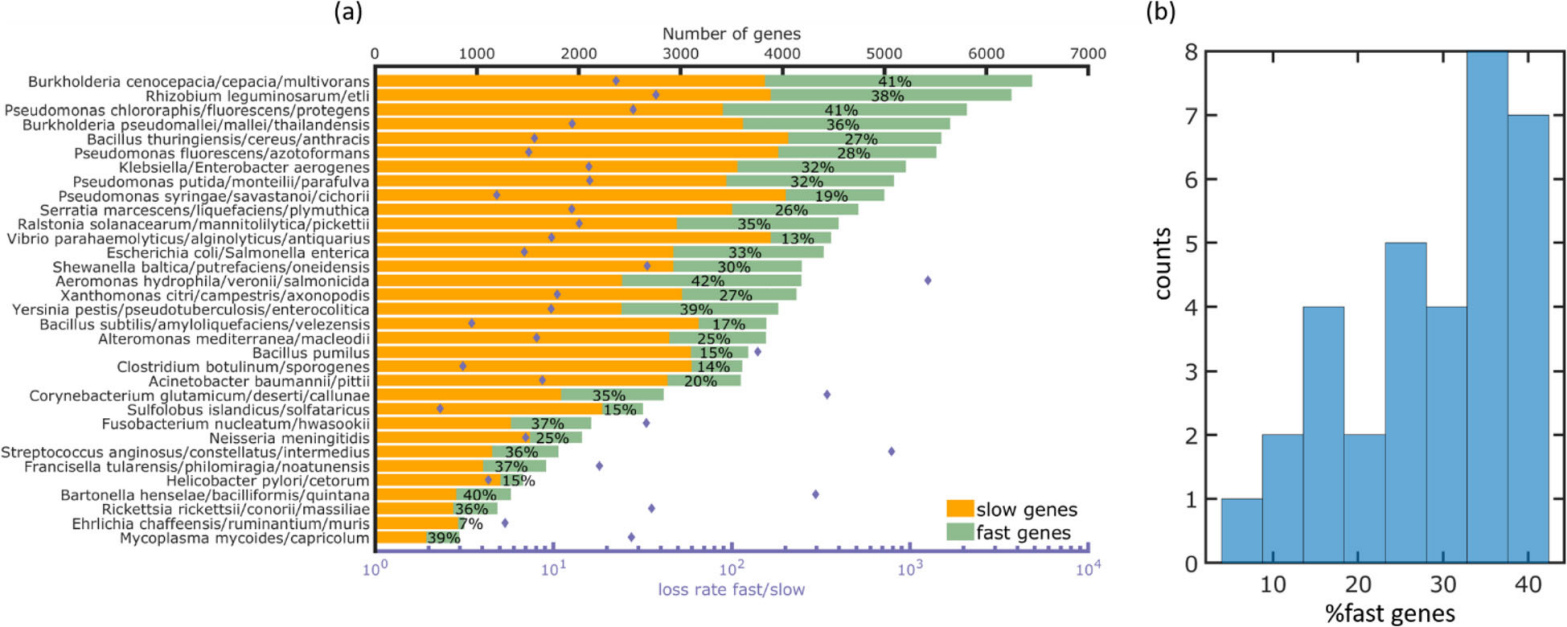
Inferred model parameters for the 33 prokaryotic clusters, as obtained by fitting the IGP-CGS model to the mean genome intersections. Fitted model parameters are listed in Additional file 1: Table S1. **a** Top *x*-axis: the number of fast-evolving and slow-evolving genes in each cluster of genomes. The percentage of fast-evolving genes is indicated for each cluster. Bottom *x*-axis: the ratio between fast and slow gene turnover rates. **b** Histogram of the percentage of fast-evolving genes across the 33 genomes clusters

The turnover rate of the fast genes is roughly 10 times greater than that of the slow genes (see Fig. 5 and Additional file 1: Table S1). In terms of genome evolution, the fast or slow turnover rates reflect the different average selective effects associated with gene deletion: losing a slow-turnover gene is associated with a substantially greater fitness cost than losing a fast-turnover gene [20]. The fraction of fast-evolving genes varies from as low as 7% up to 40%, apparently reflecting substantial differences in the pangenome dynamics among prokaryotes (Fig. 5).

**Fig. 6:**
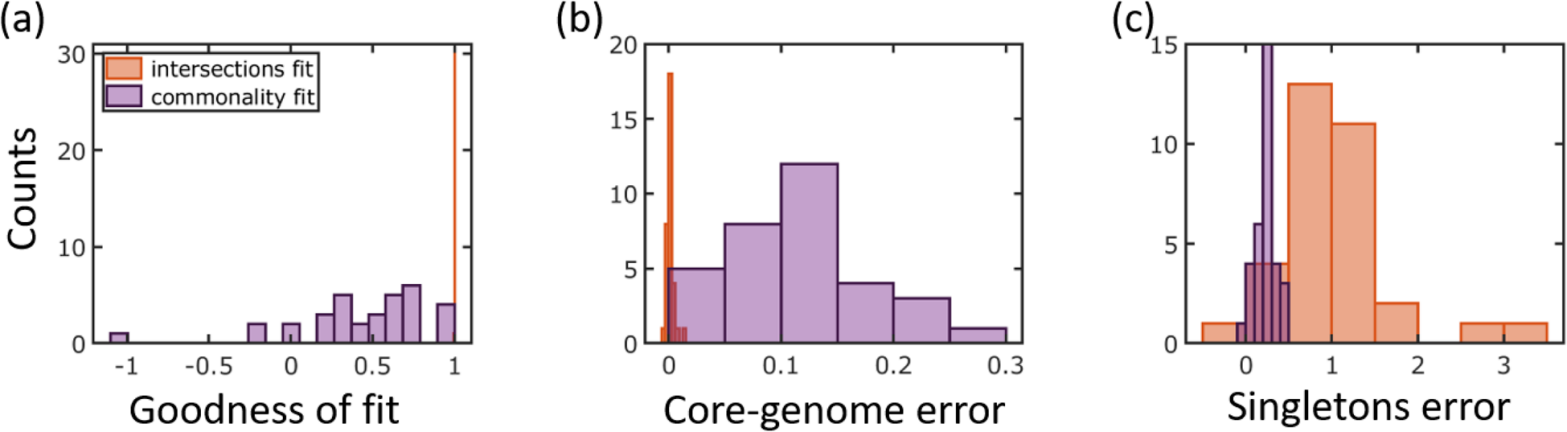
Comparison of the statistics of the IGP-CGS model fit to the 33 genomic clusters. **a** Histogram for the goodness of fit *R*^2^ to empirical mean genome intersections 〈*I*〉_*k*_. **b** Histogram for the error in core genome sizes *g*_*N*_ of model fit. The error is calculated as $\left({g}_N^{\mathrm{model}}-{g}_N^{\mathrm{data}}\right)/{g}_N^{\mathrm{data}}$. **c** Histogram of the error in model prediction for the number of singletons, as computed from the model mean genome intersections using Eq. 1. The error is calculated as in **b**

The fits of the model for the *E. coli* cluster are shown in Fig. 4. First, we fit the model directly to the gene commonality distribution, as it is usually done in the analyses of the U-shaped distributions [5–8]. Although a good fit to the U-shaped distribution can be obtained (Fig. 4), the inferred gene turnover rates are underestimated, as indicated by the poor fit obtained for the genome intersections (Fig. 4). Accordingly, the core genome size is overestimated by this fit. By contrast, when the turnover rates are inferred directly from gene intersections (Fig. 4), the core genome size is estimated accurately but the number of singletons is overestimated (Fig. 4). This discrepancy is not unique to *E. coli* and was observed across the entire analyzed set of genome clusters (Fig. 6). Specifically, under the IGP-CGS model, for all genome clusters, the goodness of fit of the model to mean intersections 〈*I*〉_*k*_ was greater than 0.995 (Fig. 6a), and the error in the core genome size was negligible (Fig. 6b), but the number of singletons was consistently overestimated (Fig. 6c). The fact that the model overestimates the number of singletons implies that fewer new genes are gained by evolving prokaryotic genomes than expected from the number of gene losses inferred directly from genome intersections. This conclusion is supported by the direct fit of the model to the gene commonality distribution (Fig. 4): in this case, gene loss rates are underestimated, and as a result, the error shifts from the number of singletons to the core genome size (Fig. 6). In addition to the mean intersections ‹*I*›_*k*_ and gene commonality *g*_*k*_, we fitted the model to the cumulative gene commonality *J*_*k*_. The cumulative gene commonality *J*_*k*_ gives the number of genes that are common to at most *k* genomes and is defined as:

4 $$ {J}_k={\sum}_{n=1}^k{g}_n $$

Comparison of the fits using the three different statistics (〈*I*〉_*k*_, *g*_*k*_, and *J*_*k*_) is shown in Additional file 1: Figs. S7-S12. Although the error in the number of singletons is smaller for *J*_*k*_ compared to ‹*I*›_*k*_, the gene turnover rates are not estimated correctly using this statistic, which is reflected in a greater error in the estimation of the core genome (see Additional file 1: Fig. S13).

Finally, we sought to determine the optimal number of gene classes and to ascertain that the overestimation of the number of singletons when fitting the IGP-CGS model to the mean intersections 〈*I*〉_*k*_ is not due to our choice to fit two classes of genes. First, because the intersection curve *I*_*k*_ as a function of the evolutionary distance *D*_*k*_ deviates from a straight line in a semi-log plot, it is evident that more than one class of genes is required to fit the genomic data. We therefore fitted the genomic data with two classes of genes and obtained a goodness of fit of 0.996 or greater. Next, we fitted the genomic data with three classes of genes and compared the 3-class fit to the 2-class fit. Although for some genome clusters the 3-class fit to the mean intersections was better than the 2-class fit, in all genome clusters, the error in the estimation of the number of singletons by the 3-class model was not reduced (see Additional file 1: Table S2). Specifically, in eight genome clusters, the 3-class fit was better than the 2-class fit in terms of adjusted *R*^2^, but in all these cases, the error in the number of singletons was greater in the three-class fit (see Additional file 1: Table S2). For example, with the three-class model, the largest improvement in the adjusted goodness of fit of 0.005 was observed for *Corynebacterium glutamicum*. The overestimation of the number of singletons was increased from 962 for the two-class fit to 1328 in the three-class fit. In three cases (*Helicobacter pylori*, *Sulfolobus islandicus*, and *Francisella tularensis*) the goodness of fit of the 3-class model was slightly higher than that of the 2-class fit; however, the adjusted goodness of fit was lower such that the additional parameters of the 3-class model are not justified by the improvement. In these three cases, the overestimation of the number of singletons was greater in the 3-class model fit (Additional file 1: Table S2). In the remaining 22 genome clusters, an attempt to fit three classes of genes effectively led to a two-class model: within the fitted parameters, either two out of the three decay constants were identical, or the size of one of the classes was close to zero.

**Fig. 7:**
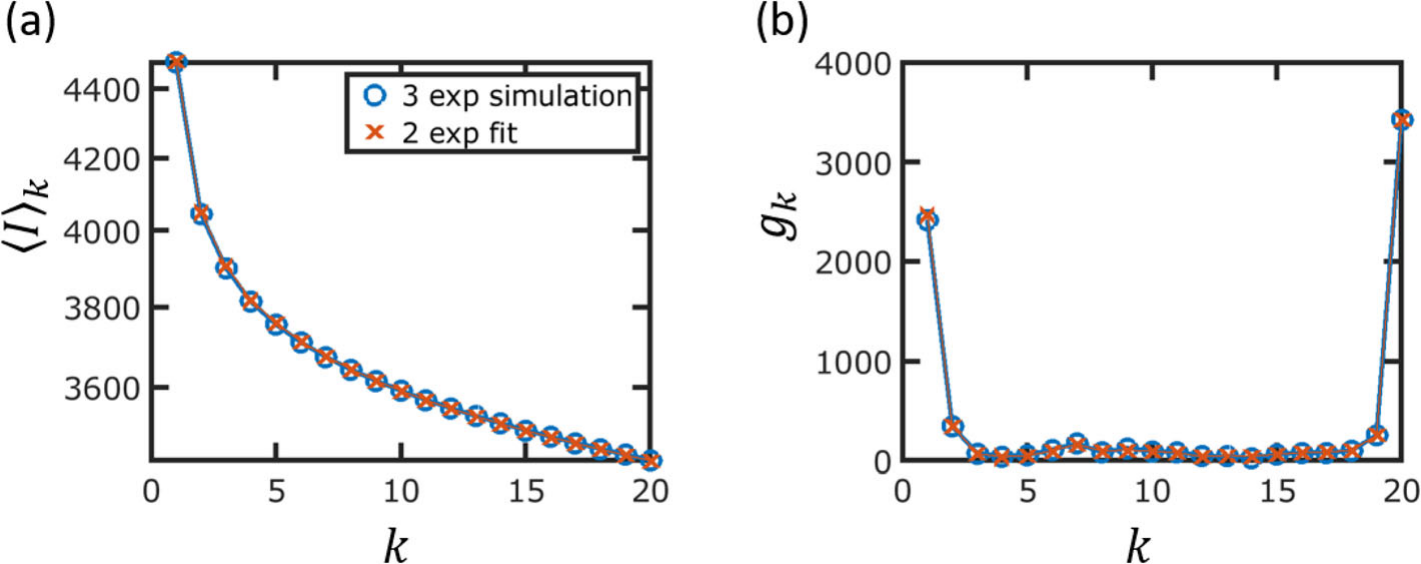
Fits of two-class model to three-class simulated dataset. **a** The mean genome intersections for a three-class simulated dataset and a two-class model fit. The slow, intermediate, and fast classes in the simulation contained 2250, 1500, and 750 genes, with *λ* values of 0.005, 0.1, and 2, respectively. The two-class fit goodness of fit is *R*^2^ = 0.99991 with 3730 slow genes and 749 fast genes, with respective *λ* values of 0.04 and 1.98. **b** Gene commonality for the simulated dataset and model prediction. The model prediction for gene commonality is extracted from the mean genome intersections of **a**, using Eq. 3

Last, we used a simulated dataset to demonstrate that fitting a two-class model to a three-class dataset will not result in an overestimation of the number of singletons. Similar to the simulations shown in Fig. 3, we simulated a dataset using the tree of the *E. coli* cluster with three classes of genes under the assumptions of an infinite gene pool and equal gene gain and gene loss rates. As shown in Fig. 7, fitting a two-class model to the mean intersections did not result in overestimation of the model prediction for the number of singletons.

The exponential decay of genome intersections was obtained under the IGP-CGS assumptions whereby, on average, any lost gene is replaced by another gene and any gained gene is new to the given set of genomes. Since we ruled out the possibility that fitting a model with three classes will improve the fit, the overestimation of the number of singletons implies the breakdown of either of these assumptions or both. In other words, either the genome size is not constant, so that genomes shrink with time, or gene regain from the external gene pool is common (the gene pool is, then, finite), or both. Incorporating either a finite gene pool or a varying genome size into the calculation of genome intersections dramatically complicates the calculations and is beyond the scope of this work. Instead, we used simulations to explore separately the effect of genome size variation (IGP-V(ariable) GS assumption) and finite gene pool (F(inite)GP-CGS) and demonstrate for *E. coli* that a good fit to the genomic data can be achieved with either of these modifications.

**Fig. 8:**
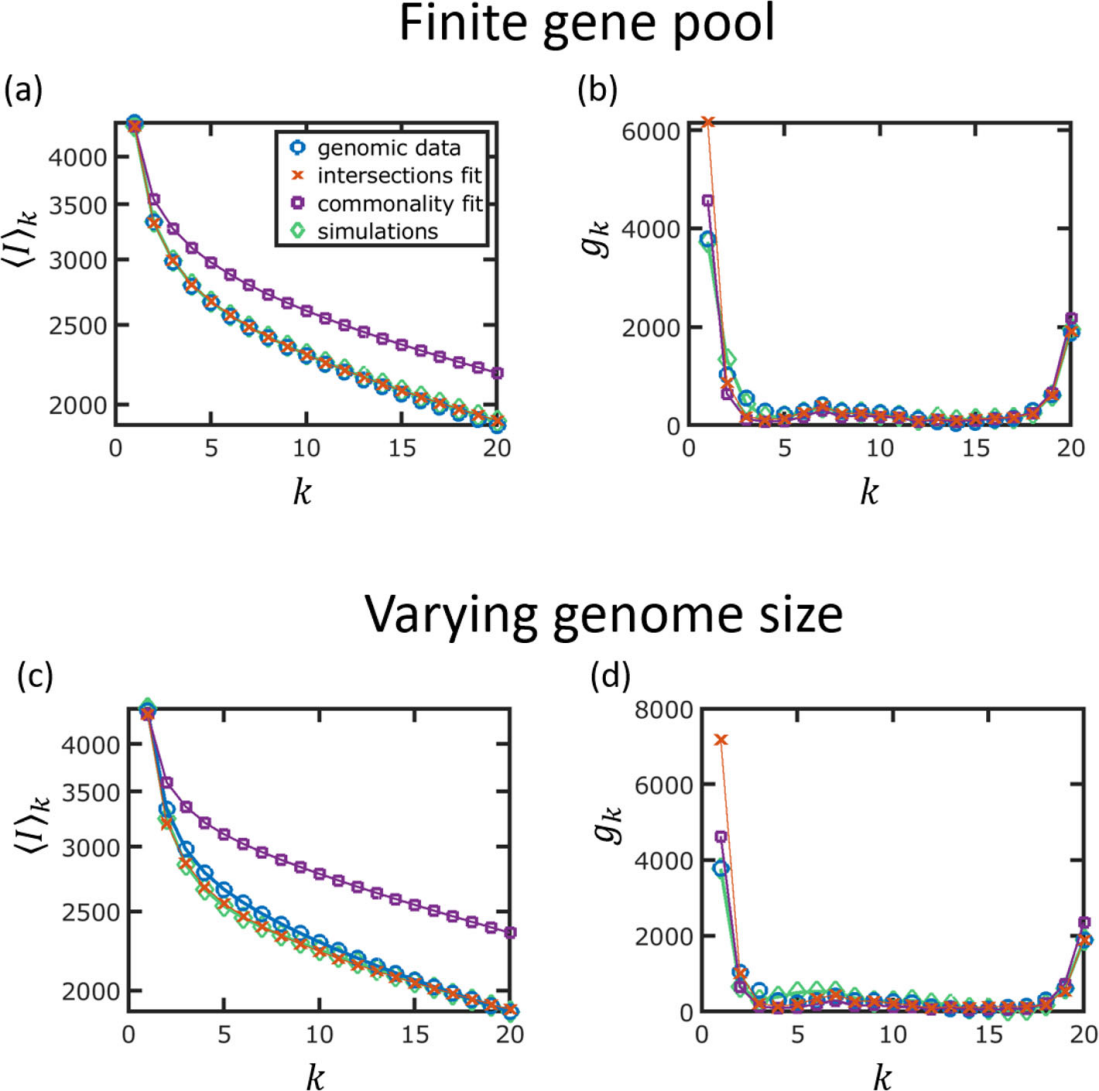
Analysis of simulated datasets for a finite gene pool (**a**, **b**) and a varying genome size (**c**, **d**). **a** The mean genome intersections for finite gene pool simulation. The parameters that were used in the simulations are the model parameters inferred under the IGP-CGS assumptions (see Additional file 1: Table S1). For slow-evolving genes, the pool size was 3 times the number of slow genes, and the fast-evolving gene pool was 9 times the number of fast genes. The IGP-CGS model fits and genomic data are also shown, as indicated in the legend. **b** Gene commonality distribution for the finite gene pool simulation, for the same model parameters as in **b**. **c** The mean genome intersections for varying genome size. For slow-evolving genes, the ratio of gain and loss rates is 1.26, whereas for the fast-evolving genes, this ratio is 0.16. **d** Gene commonality distribution for varying genome size for the same model parameters as in **c**

To simulate the evolution of the *E. coli* pangenome for a finite gene pool (FGP-CGS) or a varying genome size (IGP-VGS), we used the same simulation scheme as before (Fig. 3e–g). A finite gene pool is introduced by limiting the number of genes that are available to the evolving genomes (see the “Methods” section). The violations of either of the IGP-CGS assumptions are considered separately to reduce the dimensionality of the parameter space and thus to allow realistic computation times. Given that it is impractical to scan numerically the entire 6-dimensional parameter spaces of the FGP-CGS or IGP-VGS models, the model parameters that were inferred for *E. coli* under the IGP-CGS assumptions were taken as the starting point (Fig. 3e–g, Additional file 1: Table S1), and only a two-dimensional parameter space was scanned. For the FGP-CGS simulation, the two parameters were the sizes of the external gene pools, those for the slow- and fast-evolving genes. For the IGP-VGS simulation, the two parameters were the gain rate to loss rate ratios for the slow- and fast-evolving genes. Two examples for simulated datasets with genome intersection and gene commonality values similar to those in *E. coli* are shown in Fig. 8. Fitting the IGP-CGS model to the simulated datasets further demonstrated similarities between the simulated and genomic data. As with the genomic dataset, fitting the model directly to the gene commonality distribution resulted in the underestimation of the gene turnover rates and the ensuing overestimation of the core genome size (Fig. 8a, c). Conversely, direct inference of turnover rates from the genome intersections resulted in the overestimation of the number of singletons (Fig. 8b, d). The discrepancy in the IGP-CGS model predictions, which was observed both with the simulated and the genomic datasets, indicates that violation of either of the model assumptions is a plausible explanation for the overestimation of the number of singletons by the IGP-CGS model.

## Discussion

The analysis presented here provides a theoretical framework to quantitatively analyze prokaryotic pangenome evolution and divergence. The analysis highlights the relation between the mean genome intersection ‹*I*›_*k*_ and the gene commonality *g*_*k*_ (Eq. 3). The relation of Eq. 3 is exact and does not involve any approximation or assumption. However, the inference of genome intersection *I*_*k*_ (Eq. 8 below) is performed within several simplifying assumptions. The assumptions of the model are rooted in the empirical data of comparative genomics and hence can be considered realistic. The four central assumptions and the underlying justification can be summarized as follows:

1. New genes can be acquired only via HGT, whereas other mechanisms, such as duplication followed by divergence and de novo gene birth, are disregarded. Although each of these routes contributes to the genome dynamics in prokaryotes, reconstructions of genome evolution indicate the contribution of HGT is by far the greatest [12, 13].
2. The unit of evolution is one gene, so that acquisition and deletion of individual gene events occur independently. This is a simplification because genes are often transferred in groups, for example, in integrative and conjugative elements and other types of genomic islands [28, 29]. Nevertheless, the genomic synteny between related prokaryotes decays much faster with the evolutionary distance than gene composition [21, 30], suggesting that the assumption is valid as the first approximation
3. Gain and loss rates are similar, such that the genome size is approximately constant in the long term, or in other words, evolving prokaryotic genomes are close to equilibrium. The measured rates of gene loss and gain vary widely [13]. Nevertheless, the equilibrium assumption yields a good fit to the observed genome size distributions in clusters of closely related prokaryote genomes [24], indicating that this assumption is reasonable when applied to long-term averages. On average, the selection coefficient associated with gene gain is positive, resulting in a gain to loss ratio greater than unity (Fig. 8 and ref. [20]), but this does not violate the equilibrium assumption because the effect of selection for gene gain is offset by the intrinsic deletion bias [20].
4. All prokaryotic populations have access to an infinite gene pool, and gene exchange rates are constant in the course of evolution. An infinite gene pool can only be an idealization, but the pangenomes of most bacteria are expansive, orders of magnitude larger than the size of a typical genome [1, 13, 31]. Nevertheless, this appears to be the most shaky of the assumptions underlying the IGP-CGS model.

In addition to the four central assumptions detailed above, the model involves several other simplifying assumptions. For example, it is assumed that selection coefficients are constant, such that adaptation to ecological niches [32], fluctuating environments [33–35], and frequency-dependent selection [36] are ignored. Here, we examine only assumptions 3 and 4 above. Taking into account the other assumptions in the further development of evolutionary models should be informative for an improved understanding of the evolution of prokaryotes. It should be emphasized that we do not claim that only violation of assumptions 3 or 4 could explain the overestimation of the number of singletons by the IGP-CGS model, and alternative explanations for this discrepancy are likely to exist.

Although our model produces good fits to genome intersection and the gene commonality distributions under the IGP-CGS assumptions, the number of singletons is systematically overestimated. It should be stressed that this overestimation cannot be explained by mis-annotations of genes, e.g., either underprediction or over-prediction of poorly conserved, small genes that comprise the majority of the singletons. Prediction of more (or fewer) singletons will increase the genome size, which will affect the entire calculation, and will not improve the fit. Thus, we hypothesized that the number of singletons is overestimated due to the violation of one or both of the IGP-CGS, namely, the infinite external gene pool and/or the (statistical) equality of the gene gain and loss rate, that is, the equilibrium state of the evolving microbial genomes. To test this hypothesis, we generated and analyzed simulated datasets for either finite gene pool (FGP-CGS) or varying genome sizes (IGP-VGS), obtaining results similar to those obtained in the genomic data analysis. However, numerical scanning of the two-dimensional parameter space for both FGP-CGS and IGP-VGS models failed to identify a single set of parameters yielding the best fit (Additional file 1: Fig. S14). Furthermore, to make the computation feasible, violations of the IPG-CGS assumptions were introduced one at a time. Thus, the present analyses do not allow us to differentiate between the violations of the infinite gene pool and constant genome size assumptions. Furthermore, it appears likely that different factors are important in different groups of microbes and that, in some cases, both assumptions are violated simultaneously. Indeed, there is empirical evidence of deviations from each of these assumptions in bacterial evolution. Our previous analysis performed with the same ATGC dataset has shown that the evolution of most groups of bacteria was dominated by gene loss, the rate of which far exceeded that of gene gain [13]. Given the much higher gene loss rate in the fast gene class compared to the slow gene class, due to the weak selection on the fast genes, as shown here, it can be expected that the fast class is depleted over time. Conceivably, some of the groups of prokaryotes losing the fast genes are headed towards extinction, whereas in others, the steady gene loss is, most likely, compensated by episodes of massive gene gain, such that on a long enough time scale, the number of genes is maintained. Thus, the constant genome size assumption appears to be frequently violated. Furthermore, direct measurements of gene regain have shown conspicuous differences among prokaryotes, such that, for some bacteria, the pool available for gene acquisition appeared to be effectively infinite, whereas for others, it was found to be finite and comparatively small [13]. Comparative genomic analyses demonstrate multiple acquisitions of similar genetic elements for example in *Salmonella enterica* plasmids [37] and *Staphylococcus aureus* genetic elements [38]. Accordingly, the infinite gene pool assumption is, in the least, not universally valid either.

The breakdown of both assumptions of IGP-CGS could be framed in terms of the cost of incorporation of new genes by microbes. In both cases, the number of gained new genes (as opposed to variants of genes already represented in a genome) is smaller than that predicted by the IGP-CGS model, suggesting that the major factor that restricts the expansion of the pangenome is the capacity to successfully incorporate new genes, i.e., the HGT barrier [26]. The existence of a substantial HGT barrier is supported also by experimental analysis of the assimilation of xenologs of orthologous genes by bacteria [39]. In these experiments, replacement of an essential *E. coli* gene by xenologs resulted in a substantial drop in fitness that was partially alleviated during the subsequent evolution of the recipient bacteria in the laboratory. In the course of the long-term evolution of microbes, the HGT barrier is likely to be broken episodically as a result of major changes in environmental conditions when extensive HGT favors survival [13, 40]. Different functional classes of microbial genes show substantial differences in evolutionary plasticity or, in other words, are differentially affected by the HGT barrier [20]. Thus, analysis of pangenome dynamics separately for each class can be expected to reveal the interplay between the key factors of genome evolution.

## Conclusions

Here, we present a theoretical framework for quantitative analysis of the evolutionary dynamics of the gene frequencies in microbial pangenomes. Specifically, we infer gene turnover rates from genome intersections and reconstruct the U-shaped distribution that is observed for gene commonality for a genomic dataset consisting of 33 groups of closely related prokaryotic genomes. The asymmetrical U shape of the gene commonality distribution is extremely general, effectively, a universal of genome evolution, being observed for genomes at all phyletic depths, from a single species to the entirety of bacteria and archaea [4, 7]. By contrast, the pangenome size is not a well-defined characteristic, being sensitive to the number of genomes in a cluster, genome sampling, and the tree depth [27]. It is therefore unclear what can be considered a “large” or a “small” pangenome, and comparison of the pangenome sizes for different organisms is not a straightforward task. Our analysis implies that, to attain insights into the evolutionary dynamics of microbes, it is preferable to measure and compare gene turnover rates that are more robust to the number and sampling of the analyzed genomes than the pangenome size. For 25 of the 33 genome clusters we analyzed, a model with two classes of genes yields a better fit of the observed gene commonality distribution than a model with three classes of genes. Thus, the genes in prokaryote genomes can be roughly divided into two categories, fast-evolving and slow-evolving. Finally, the overestimation of the number of singletons by the IGP-CGS model indicates that either infinite gene pool is not a good approximation, or the gene gain lags behind the loss, or both. In either case, genome evolution in prokaryotes is limited by the availability of genes that can be acquired and retained.

## Methods

### Model for genome intersections evolution

The evolution of the genome size can be modeled as a random process, where genes are gained and lost stochastically, with rates *P*^+^ and *P*^−^, respectively. Accordingly, the dynamics of the number of genes *x* is given by the following equation [24]:

5 $$ \frac{dx}{dt}={P}^{+}-{P}^{-} $$

In the weak mutation limit, the gain and loss rates of Eq. 5 above, can be written as in Eqs. 1 and 2, where the fixation probability *F* is given by the Kimura formula [41]:

6 $$ F(S)=\frac{S}{1-{e}^{-S}} $$

and *S* is the selection coefficient associated with the acquisition of one gene, wherein principle *S* can be either positive or negative. It should be emphasized that here, only the loss rate is evaluated and that the current analysis does not allow evaluation of the selection coefficient *S*. Disentangling the effects of deletion rate from those of selection (see Eq. 2) is not trivial. This problem is addressed in our previous work [42].

For the IGP-CGS model, genome intersection of *k* genomes is given by [19]

7 $$ {I}_k\left({D}_k\right)=x{\mathrm{e}}^{-\uplambda {D}_k} $$

where the decay constant is given by the per-gene loss rate *λ* = *P*^−^/*x*, and *D*_*k*_ is the total evolutionary distance spanned by those *k* genomes. Specifically, *D*_*k*_ is given by the sum of all branch lengths in the phylogenetic tree that describes the evolutionary relations of the *k* genomes. Analyses of the genomic data imply that genomes are composed of slow- and fast-evolving genes [21]. Thus:

8 $$ {I}_k\left({D}_k\right)={x}_1{\mathrm{e}}^{-{\uplambda}_1{D}_k}+{x}_2{\mathrm{e}}^{-{\uplambda}_2{D}_k} $$

with *x*_1_ and *x*_2_ being the average numbers of genes in each class. Fitting the data to the model requires therefore inference of four parameters: *x*_1_, *x*_2_, *λ*_1_, and *λ*_2_.

For a cluster of *N* genomes, there are ${C}_k^N$ subsets of *k* genomes, where ${C}_k^N$ denotes the binomial coefficient. To extract gene commonality from genome intersections using Eq. 3, we wish to evaluate the mean intersection for each *k*, denoted 〈*I*〉_*k*_. The first stage is to obtain all possible ${C}_k^N$ phylogenetic subtrees that include *k* genomes, by pruning the tree of the complete set of *N* genomes. Next, the sum of the branch lengths for each *k* genomes subtree is evaluated to construct the probability density function *P*(*D*_*k*_) to observe the sum of branch lengths between *D*_*k*_ and *D*_*k*_ + d*D*_*k*_. Finally, the mean intersection 〈*I*〉_*k*_ is given by a weighted average over branch lengths:

9 $$ {\left\langle I\right\rangle}_k=\int {I}_k\left({D}_k\right)\ P\left({D}_k\right)\ \mathrm{d}{D}_k $$

where *I*_*k*_(*D*_*k*_) is given by either Eq. 7 or Eq. 8. Note that *P*(*D*_*k*_) reflects both, the phylogenetic tree topology and the branch lengths.

### Simulation scheme

A cluster of genomes is simulated along a tree, using the Gillespie simulation scheme [43]. A genome is represented as a collection of genes, and possible mutations are either gene gain or gene loss of rates *P*^+^ and *P*^−^, respectively. The occurrence of mutations therefore follows a Poisson distribution with parameter *a*_0_, which is given by:

10 $$ {a}_0={P}^{+}+{P}^{-} $$

The waiting time between mutation events *τ* follows an exponential distribution with rate parameter *a*_0_. At each simulation, step *τ* is picked from an exponential distribution, using:

11 $$ \tau =-\ln (r)/{a}_0 $$

where *r* is a random number drawn from a uniform distribution between 0 and 1. After determining the waiting time, it is determined whether a gene is gained or lost at random, according to *P*^+^ and *P*^−^. Under the IGP-CGS assumptions, the simulation scheme takes as an input in addition to the tree two parameters: initial genome size *x* and gene loss rate *P*^−^ (which is under the CGS assumption also determines the gain rate *P*^+^).

Empirical genome intersections imply that two gene turnover rates are required to fit the genomic data (see Eq. 8). To generate more realistic datasets, genomes containing two classes of genes, fast- and slow-evolving genes, are simulated. In addition to the tree, this simulation scheme includes four parameters: *x*_1_, *x*_2_, and loss rates for slow- and fast-evolving genes, ${P}_1^{-}$ and ${P}_2^{-}$. Extending the simulation scheme to account for the breakdown of either of the IGP-CGS assumptions will add two parameters, to a total of six. For the FGP-CGS assumption, the total number of genes that are available to the evolving genomes is set to a finite number. Fast and slow genes evolve independently and are assumed to be drawn from two different pools, such that the two additional parameters are the gene pool sizes, *L*_1_ and *L*_2_. Under the IGP-VGS assumption, the gain and loss rates are not necessarily equal, which add two additional parameters, ${P}_1^{+}$ and ${P}_2^{+}$.

### Genomic dataset

We used the Alignable Tight Genomic Clusters (ATGC) database [44] to compile 33 groups of closely related prokaryotic genome. The dataset includes 32 groups (or ATGCs) of bacteria and one group of archaea (see Additional file 1: Table S1 for the list of ATGCs analyzed in this study). The selected ATGCs meet the following criteria: (i) maximum pairwise tree distance is at least 0.1 substitutions per site and (ii) the phylogenetic tree contains more than two clades, such that pairwise tree distances are centered around more than two typical values. To allow reasonable computational times, ATGCs with more than 20 genomes were sampled such that each ATGC contains at most 20 representative genomes. Throughout the analysis, phylogenetic trees were rescaled to compensate for the systematic underestimation of branch lengths that is apparently due to homologous recombination [19].

## Supplementary Information

**Additional file 1: Table S1**. Genome clusters (ATGCs) in the analyzed dataset and model parameters that were inferred under the IGP-CGS assumptions. **Table S2.** Comparison of 2-class and 3-class model fits. Goodness of fit *R*^2^, adjusted goodness of fit ${R}_{adj}^2$, and the difference between model prediction for the number of singletons and the actual value ∆*g*_1_ are listed. Eight cases where the 3-class fit is better than the 2-class fit in terms of ${R}_{adj}^2$ are highlighted. In all cases, the error in the 3-class model prediction for the number of singletons is greater or equal to the error of the 2-class model prediction. **Figure S1.** Genome intersections and gene commonality distribution for the analyzed genomic dataset. The IGP-CGS model fits are also indicated, see legend of Fig. 4 in the main text. Each row shows a different cluster, which is indicated in the row heading. **Figures S2-S6.** Same as Fig. S1. **Figure S7.** Genome intersections, gene cumulative commonality and gene commonality distribution for the analyzed genomic dataset. The IGP-CGS model fits are indicated, as shown in the upper left panel legend. **Figures S8-S12.** Same as Fig. S1. **Figure S13.** Comparison of the statistics of the IGP-CGS model fit to the 33 genomic clusters, when fitted to mean intersections 〈*I*〉_*k*_, gene commonality *g*_*k*_, and gene cumulative commonality *J*_*k*_. a) Histogram for the error in core genome sizes *g*_*N*_ of model fit. The error is calculated as $\left({g}_N^{\mathrm{model}}-{g}_N^{\mathrm{data}}\right)/{g}_N^{\mathrm{data}}$. b) Histogram of the error in model prediction for the number of singletons, as computed from model mean genomes intersections using Eq. 3. The error is calculated as in panel a. **Figure S14.** The similarity of simulated datasets to the genomic data of ATGC001. Simulations for different pool sizes under the FGP-CGS assumption are shown in panels a-d. Simulations for different gain to loss ratios under the IGP-VGS assumption are shown in panels e-h. The similarity between the simulated data is quantified by the error in the number of singletons (panels a and e), the error in the pangenome size (panels b and f), a combined measure of the number of singletons and the pangenome size (panels c and g), and the goodness of fit for the mean intersections (panels d and h). the error *dX* is calculated as (*X*_model_ − *X*_data_)/*X*_data_. Contour lines of the optimal combined measure are indicated in panels c and g. For comparison, the optimal region in terms of the combined measure is also shown in panels d and h. The parameters that were used in the simulations that are shown in Fig. 8 of the main text are indicated by a vertical and a horizontal black lines in all panels.

## Data Availability

Thirty-three groups of closely related prokaryotic genome were compiled using the Alignable Tight Genomic Clusters (ATGC) database [31]. The 33 ATGCs analyzed in this study are listed in Additional file 1: Table S1. MATLAB code to generate and analyze the simulated dataset is publicly available at https://github.com/selait/ProkaryoticPangenome [45]. The same scripts were used to analyze the genomic dataset.

## Abbreviations

HGT: Horizontal gene transfer
ATGC: Alignable Tight Genomic Clusters database
IGP-CGS: Infinite gene pool and constant genome size
IGP-VGS: Infinite gene pool and variable genome size
FGP-CGS: Finite genome size and constant genome size
