# Supplementary material for: Assessment of assumptions underlying models of prokaryotic pangenome evolution
Source: BMC Biol. 2021 Feb 10;19:27. doi: 10.1186/s12915-021-00960-2 (PMC7874442; doi:10.1186/s12915-021-00960-2)
Supplement: Supplementary file 1 — Additional file 1: Table S1. Genome clusters (ATGCs) in the analyzed dataset and model parameters that were inferred under the IGP-CGS assumptions. Table S2. Comparison of 2-class and 3-class model fits. Goodness of fit R2, adjusted goodness of fit ${R}_{adj}^2$Radj2, and the difference between model prediction for the number of singletons and the actual value ∆g1 are listed. Eight cases where the 3-class fit is better than the 2-class fit in terms of ${R}_{adj}^2$Radj2 are highlighted. In all cases, the error in the 3-class model prediction for the number of singletons is greater or equal to the error of the 2-class model prediction. Figure S1. Genome intersections and gene commonality distribution for the analyzed genomic dataset. The IGP-CGS model fits are also indicated, see legend of Fig. 4 in the main text. Each row shows a different cluster, which is indicated in the row heading. Figures S2-S6. Same as Fig. S1. Figure S7. Genome intersections, gene cumulative commonality and gene commonality distribution for the analyzed genomic dataset. The IGP-CGS model fits are indicated, as shown in the upper left panel legend. Figures S8-S12. Same as Fig. S1. Figure S13. Comparison of the statistics of the IGP-CGS model fit to the 33 genomic clusters, when fitted to mean intersections 〈I〉k, gene commonality gk, and gene cumulative commonality Jk. a) Histogram for the error in core genome sizes gN of model fit. The error is calculated as [file 12915_2021_960_MOESM1_ESM.pdf]

Additional file 1

Theory of prokaryotic pangenome evolution

Itamar Sela, Yuri I. Wolf, and Eugene V. Koonin

Genomes names	ATGC	# genomes	# slow genes	# fast genes	P^{-}_{slow}	P^{-}_{fast}
Escherichia coli/Salmonella enterica	ATGC001	20	2929	1471	563	3877
Klebsiella/Enterobacter aerogenes	ATGC002	20	3558	1648	502	7962
Bacillus thuringiensis/cereus/anthracis	ATGC014	20	4057	1497	519	4081
Bacillus subtilis/amyloliquefaciens/velezensis	ATGC015	20	3178	658	195	679
Mycoplasma mycoides/capricolum	ATGC035	17	504	319	12	341
Rickettsia rickettsii/conorii/massiliae	ATGC044	15	768	429	581	20706
Ehrlichia chaffeensis/ruminantium/muris	ATGC045	14	814	62	102	550
Helicobacter pylori/cetorum	ATGC050	20	1232	222	187	812
Corynebacterium glutamicum/deserti/callunae	ATGC069	14	1828	1002	2	625
Pseudomonas putida/monteilii/parafulva	ATGC071	20	3452	1636	278	4457
Pseudomonas syringae/savastanoi/cichorii	ATGC075	10	4031	965	1343	6464
Pseudomonas fluorescens/azotoformans	ATGC076	11	3958	1552	975	7120
Pseudomonas chlororaphis/fluorescens/protegens	ATGC078	16	3418	2388	308	8651
Clostridium botulinum/sporogenes	ATGC081	14	3112	488	1477	4604
Burkholderia pseudomallei/mallei/thailandensis	ATGC088	20	3612	2028	357	4552
Burkholderia cenocepacia/cepacia/multivorans	ATGC089	19	3829	2618	501	11276
Sulfolobus islandicus/solfataricus	ATGC093	20	2230	397	792	1835
Serratia marcescens/liquefaciens/plymuthica	ATGC098	19	3505	1235	228	2895
Vibrio parahaemolyticus/alginolyticus/antiquarius	ATGC110	12	3886	587	65	637
Aeromonas hydrophila/veronii/salmonicida	ATGC111	15	2426	1757	2	3062
Shewanella baltica/putrefaciens/oneidensis	ATGC120	14	2929	1259	66	2217
Rhizobium leguminosarum/etli	ATGC123	10	3883	2362	217	8186
Yersinia pestis/pseudotuberculosis/enterocolitica	ATGC127	20	2420	1535	234	2270
Xanthomonas citri/campestris/axonopodis	ATGC134	13	3014	1119	130	1368
Neisseria meningitidis	ATGC137	15	1521	510	1230	8592
Francisella tularensis/philomiragia/noatunensis	ATGC138	20	1062	617	53	954
Acinetobacter baumannii/pittii	ATGC149	20	2874	715	94	818
Ralstonia solanacearum/mannitolilytica/pickettii	ATGC188	10	2963	1587	837	11668
Alteromonas mediterranea/macleodii	ATGC190	14	2889	943	179	1441
Bartonella henselae/bacilliformis/quintana	ATGC201	12	799	535	1	236
Fusobacterium nucleatum/hwasookii	ATGC261	13	1338	782	106	3503
Bacillus pumilus	ATGC290	10	3102	560	3	435
Streptococcus anginosus/constellatus/intermedius	ATGC296	11	1152	647	1	908

TABLE S1: Genome clusters (ATGCs) in the analyzed dataset and model parameters that were inferred under the IGP-CGS assumptions.

Genomes names	ATGC	2-class R^2	2-class R^2_{adj}	2-class Δg_1	3-class R^2	3-class R^2_{adj}	3-class Δg_1
Escherichia coli/Salmonella enterica	ATGC001	0.999957	0.999952	2771	0.999957	0.999946	2771
Klebsiella/Enterobacter aerogenes	ATGC002	0.999893	**0.999881**	**2714**	0.999960	**0.999949**	**3010**
Bacillus thuringiensis/cereus/anthracis	ATGC014	0.999924	0.999915	2505	0.999924	0.999904	2505
Bacillus subtilis/amyloliquefaciens/velezensis	ATGC015	0.999865	0.999849	1291	0.999865	0.999829	1291
Mycoplasma mycoides/capricolum	ATGC035	0.999635	0.999583	502	0.999635	0.999513	502
Rickettsia rickettsii/conorii/massiliae	ATGC044	0.999808	0.999777	384	0.999808	0.999732	384
Ehrlichia chaffeensis/ruminantium/muris	ATGC045	0.999985	0.999982	58	0.999985	0.999978	58
Helicobacter pylori/cetorum	ATGC050	0.999945	0.999938	691	0.999946	0.999932	696
Corynebacterium glutamicum/deserti/callunae	ATGC069	0.995957	**0.995222**	**962**	0.999915	**0.999878**	**1328**
Pseudomonas putida/monteilii/parafulva	ATGC071	0.999880	**0.999866**	**3885**	0.999941	**0.999926**	**4107**
Pseudomonas syringae/savastanoi/cichorii	ATGC075	0.999993	0.999991	494	0.999993	0.999988	494
Pseudomonas fluorescens/azotoformans	ATGC076	0.999885	0.999856	2275	0.999885	0.999808	2275
Pseudomonas chlororaphis/fluorescens/protegens	ATGC078	0.999880	0.999861	2102	0.999880	0.999836	2102
Clostridium botulinum/sporogenes	ATGC081	0.999865	0.999841	1128	0.999865	0.999805	1128
Burkholderia pseudomallei/mallei/thailandensis	ATGC088	0.999674	0.999636	2715	0.999674	0.999587	2715
Burkholderia cenocepacia/cepacia/multivorans	ATGC089	0.999870	0.999854	5515	0.999870	0.999833	5515
Sulfolobus islandicus/solfataricus	ATGC093	0.999943	0.999936	1502	0.999944	0.999929	1522
Serratia marcescens/liquefaciens/plymuthica	ATGC098	0.999707	0.999671	1866	0.999707	0.999624	1866
Vibrio parahaemolyticus/alginolyticus/antiquarius	ATGC110	0.999918	**0.999900**	**1005**	0.999964	**0.999943**	**1130**
Aeromonas hydrophila/veronii/salmonicida	ATGC111	0.999472	**0.999384**	**1358**	0.999925	**0.999896**	**1552**
Shewanella baltica/putrefaciens/oneidensis	ATGC120	0.999896	0.999877	2140	0.999896	0.999850	2140
Rhizobium leguminosarum/etli	ATGC123	0.999790	0.999731	2456	0.999790	0.999623	2456
Yersinia pestis/pseudotuberculosis/enterocolitica	ATGC127	0.999859	0.999843	2045	0.999859	0.999822	2045
Xanthomonas citri/campestris/axonopodis	ATGC134	0.999837	0.999804	889	0.999837	0.999755	889
Neisseria meningitidis	ATGC137	0.999752	0.999710	700	0.999752	0.999652	700
Francisella tularensis/philomiragia/noatunensis	ATGC138	0.999873	0.999858	1101	0.999873	0.999840	1109
Acinetobacter baumannii/pittii	ATGC149	0.999874	**0.999860**	**2515**	0.999891	**0.999862**	**2577**
Ralstonia solanacearum/mannitolilytica/pickettii	ATGC188	0.999997	0.999996	-4	0.999997	0.999994	-4
Alteromonas mediterranea/macleodii	ATGC190	0.999811	0.999776	1857	0.999811	0.999726	1857
Bartonella henselae/bacilliformis/quintana	ATGC201	0.999768	**0.999717**	**113**	0.999983	**0.999973**	**151**
Fusobacterium nucleatum/hwasookii	ATGC261	0.999712	0.999654	1040	0.999712	0.999568	1040
Bacillus pumilus	ATGC290	0.999601	**0.999487**	**566**	0.999798	**0.999637**	**609**
Streptococcus anginosus/constellatus/intermedius	ATGC296	0.999738	0.999673	587	0.999738	0.999564	587

TABLE S2: Comparison of 2-class and 3-class model fits. Goodness of fit R^2, adjusted goodness of fit R^2_{adj}, and the difference between model prediction for the number of singletons and the actual value Δg_1 are listed. Eight cases where the 3-class fit is better than the 2-class fit in terms of R^2_{adj} are highlighted. In all cases, the error in the 3-class model prediction for the number of singletons is greater or equal to the error of the 2-class model prediction.

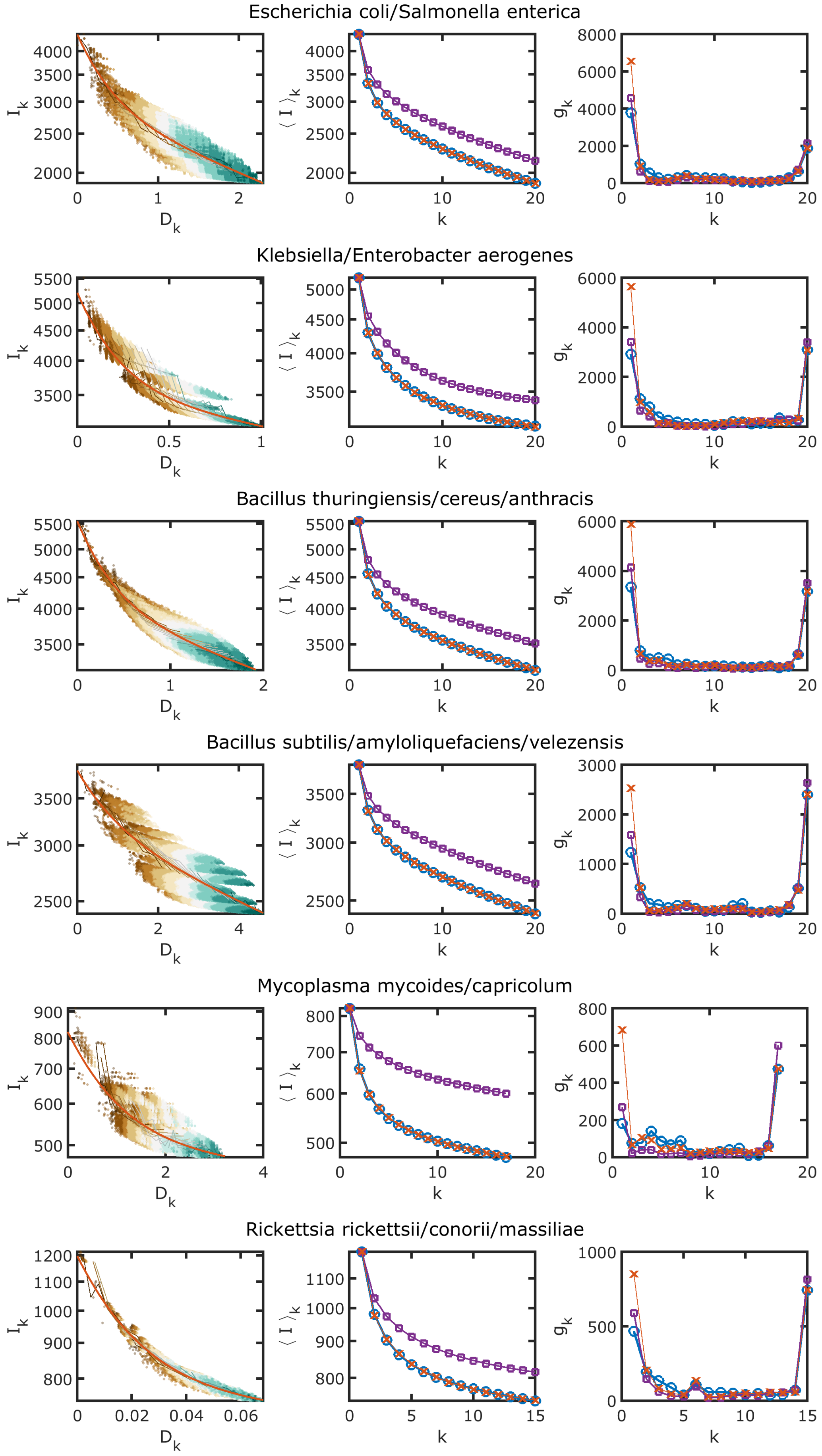

FIG. S1: Genome intersections and gene commonality distribution for the analyzed genomic dataset. The IGP-CGS model fits are also indicated, see legend of Fig. 4 in the main text. Each row shows a different cluster, which is indicated in the row heading.

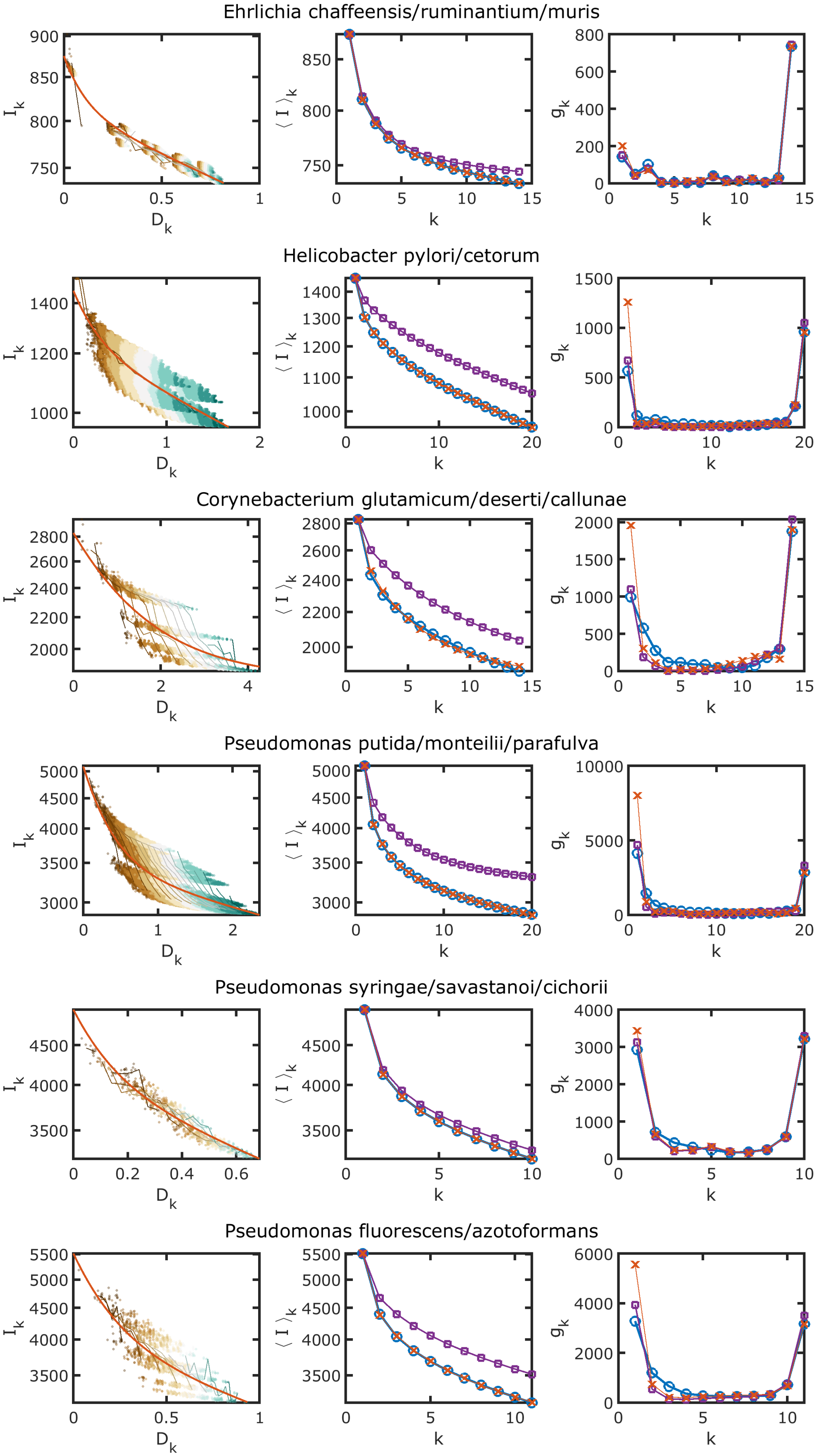

FIG. S2: Same as Fig. S1.

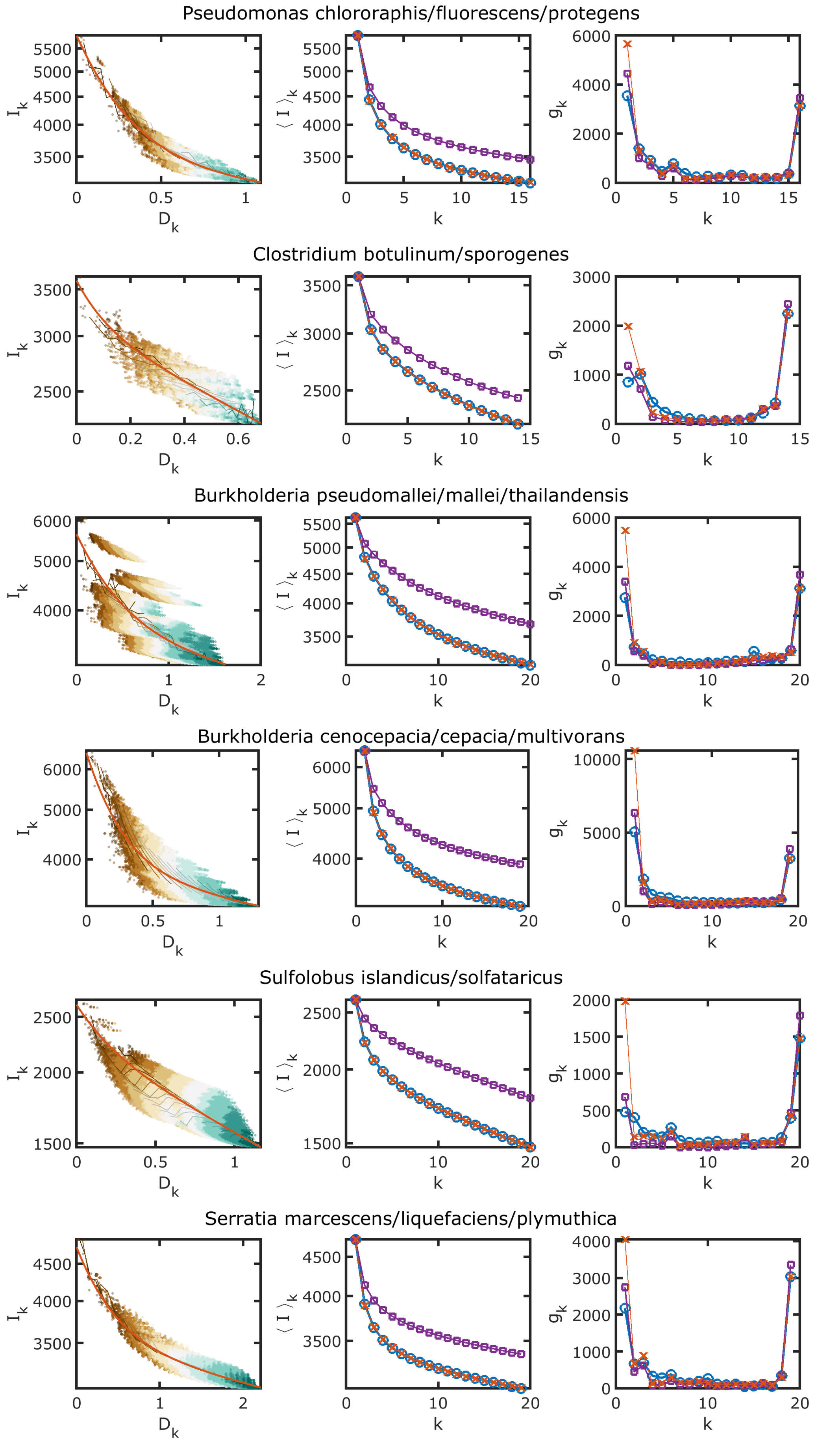

FIG. S3: Same as Fig. S1.

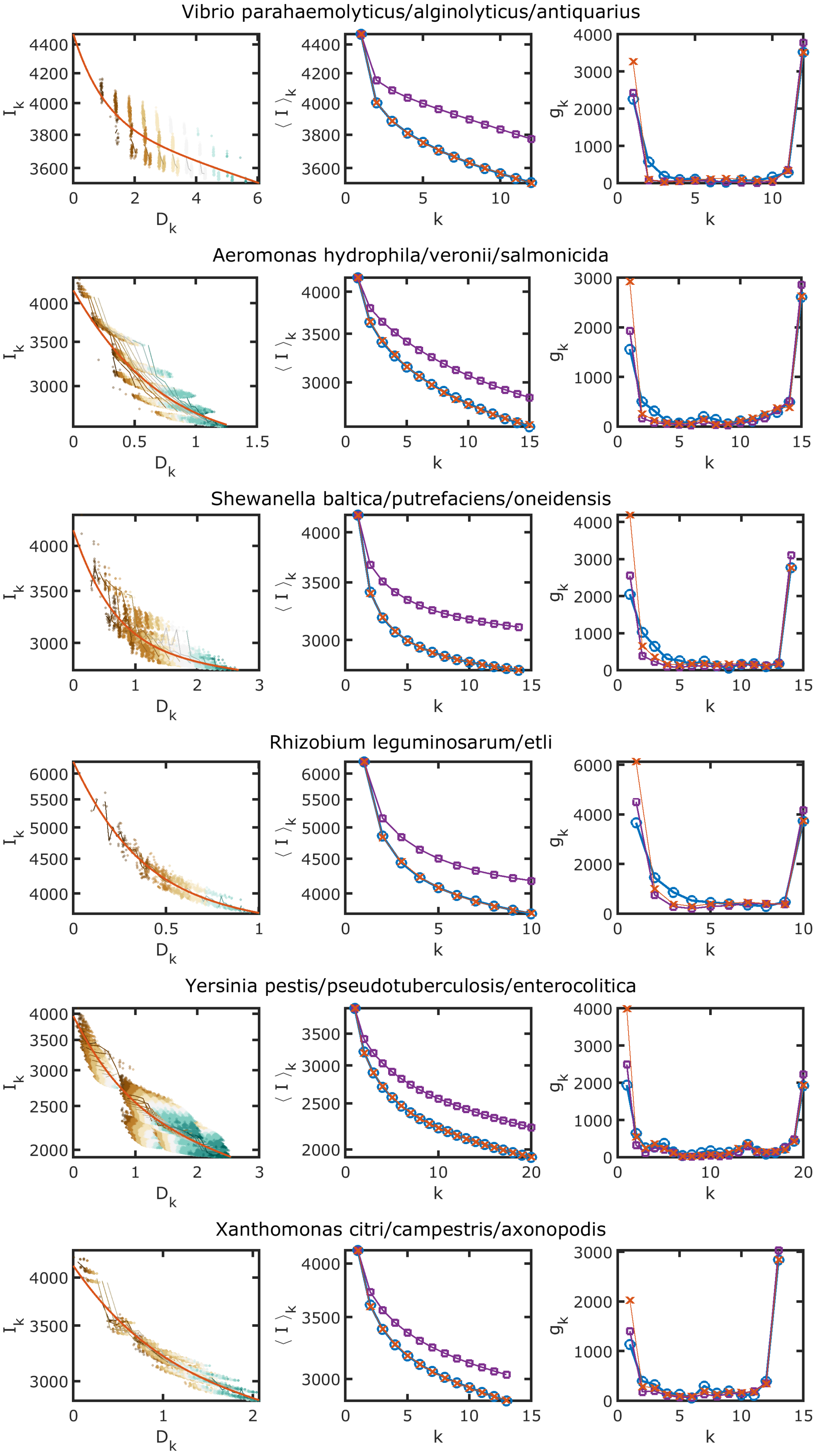

FIG. S4: Same as Fig. S1.

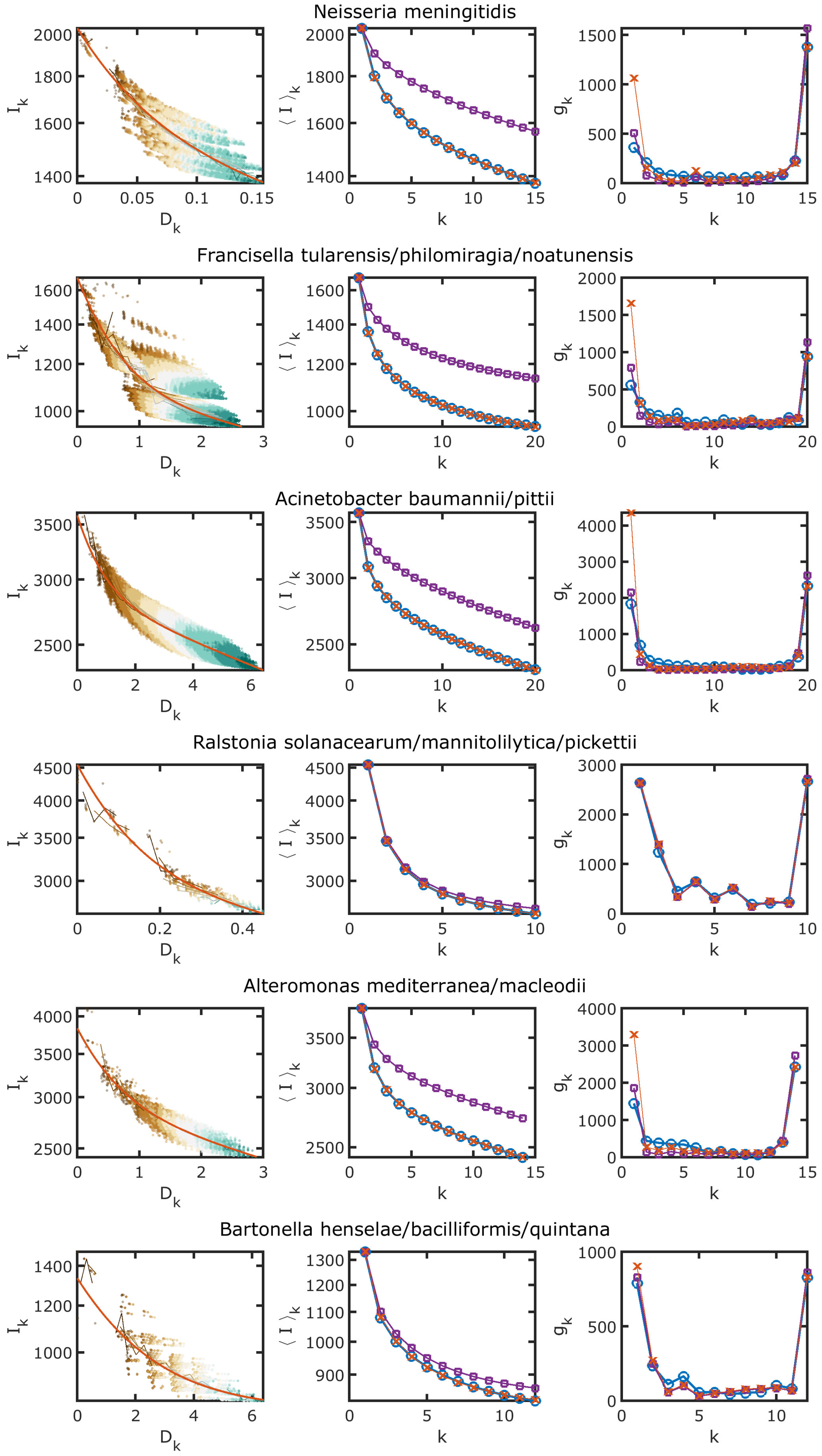

FIG. S5: Same as Fig. S1.

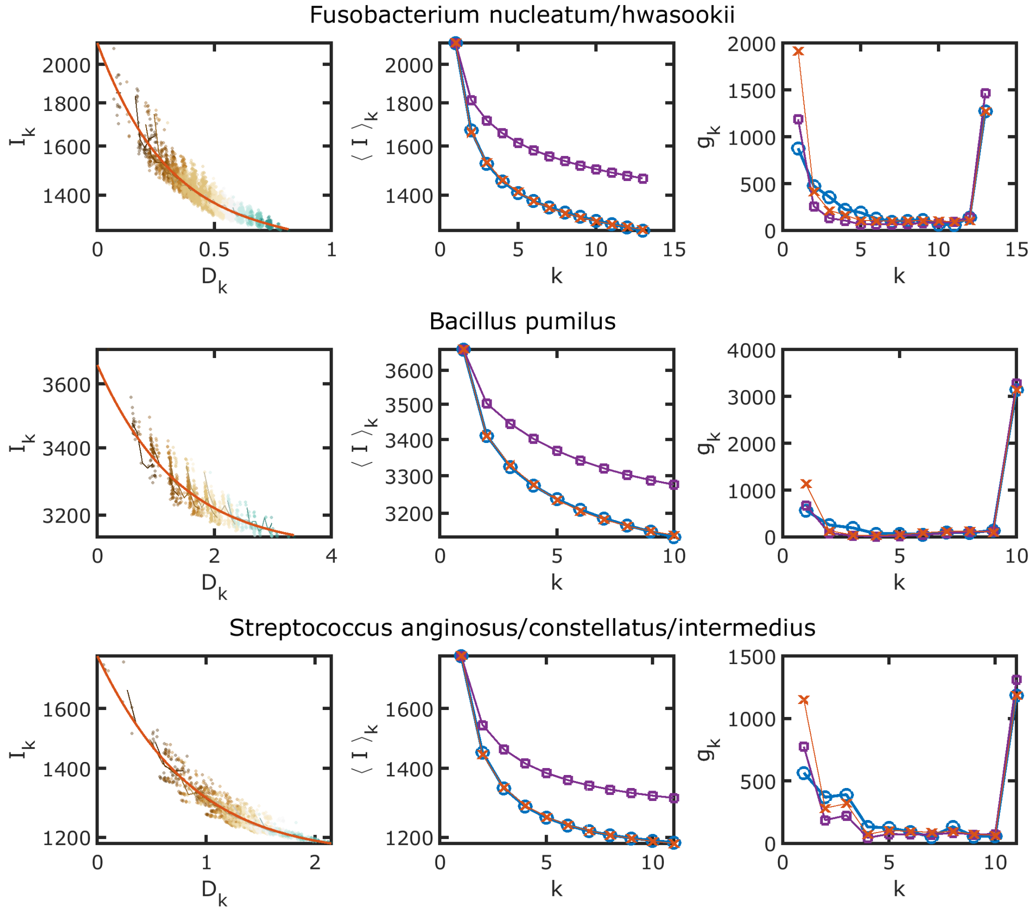

FIG. S6: Same as Fig. S1.

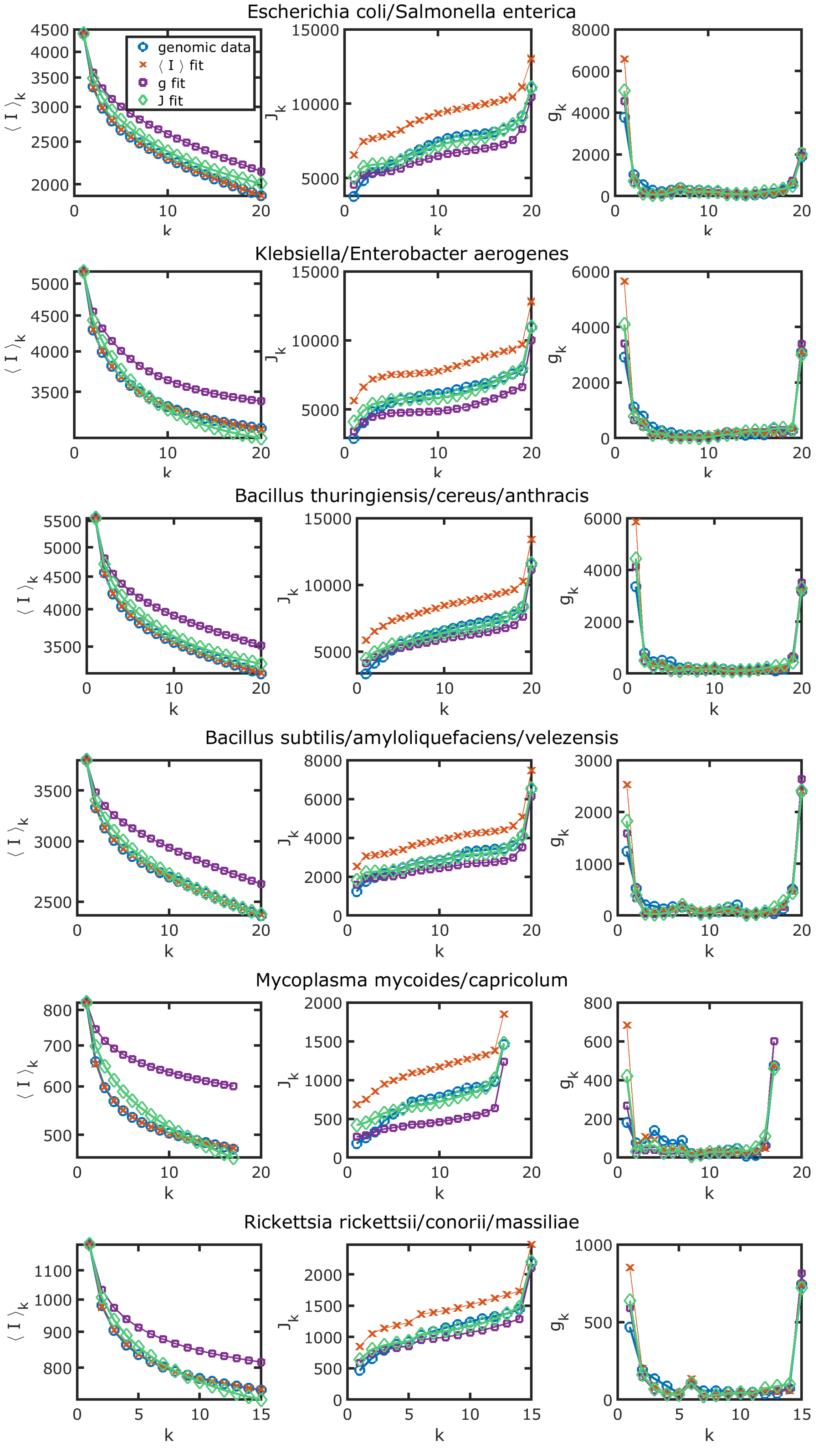

FIG. S7: Genome intersections, gene cumulative commonality and gene commonality distribution for the analyzed genomic dataset. The IGP-CGS model fits are indicated, as shown in the upper left panel legend.

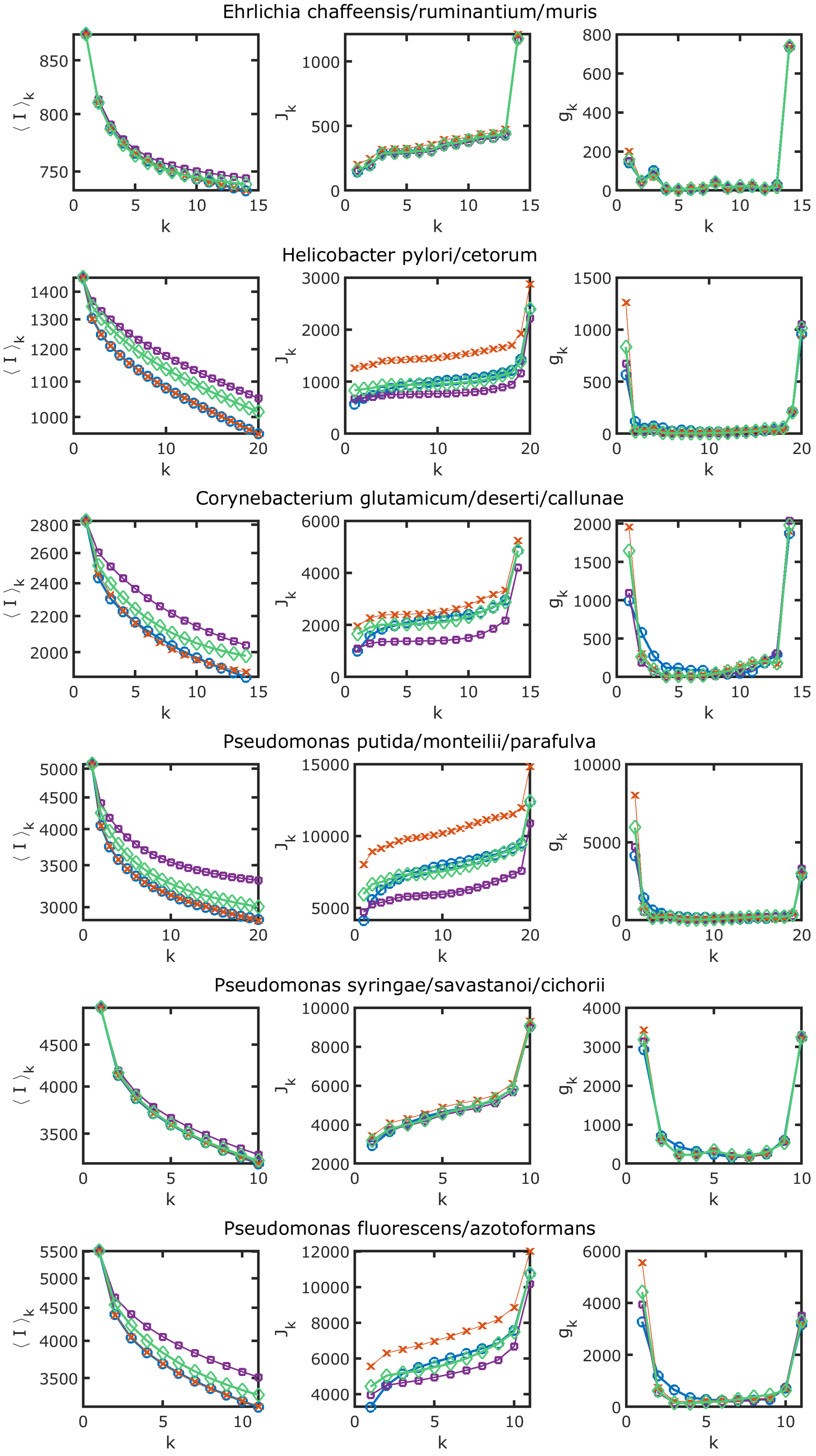

FIG. S8: Same as Fig. S7.

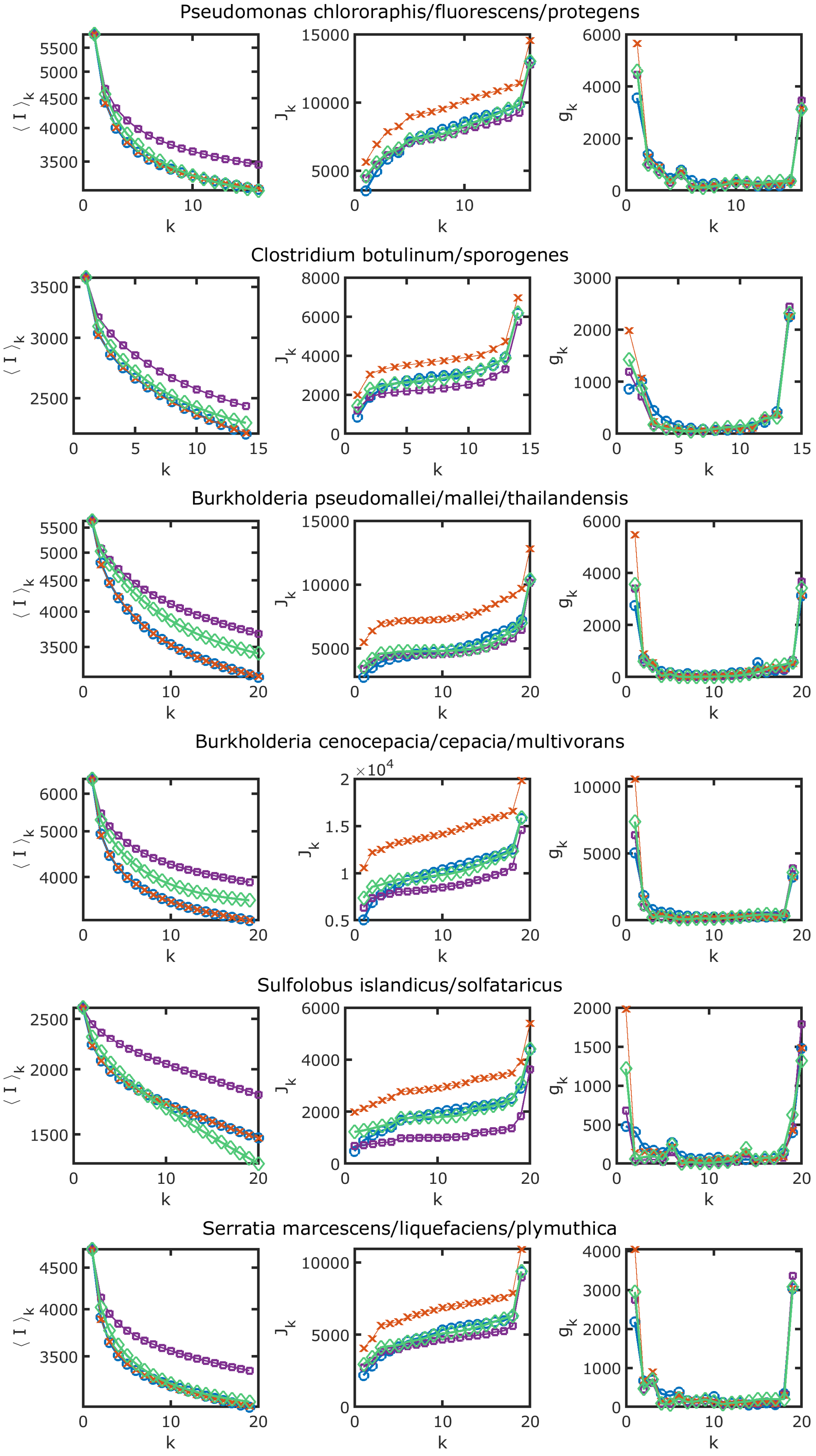

FIG. S9: Same as Fig. S7.

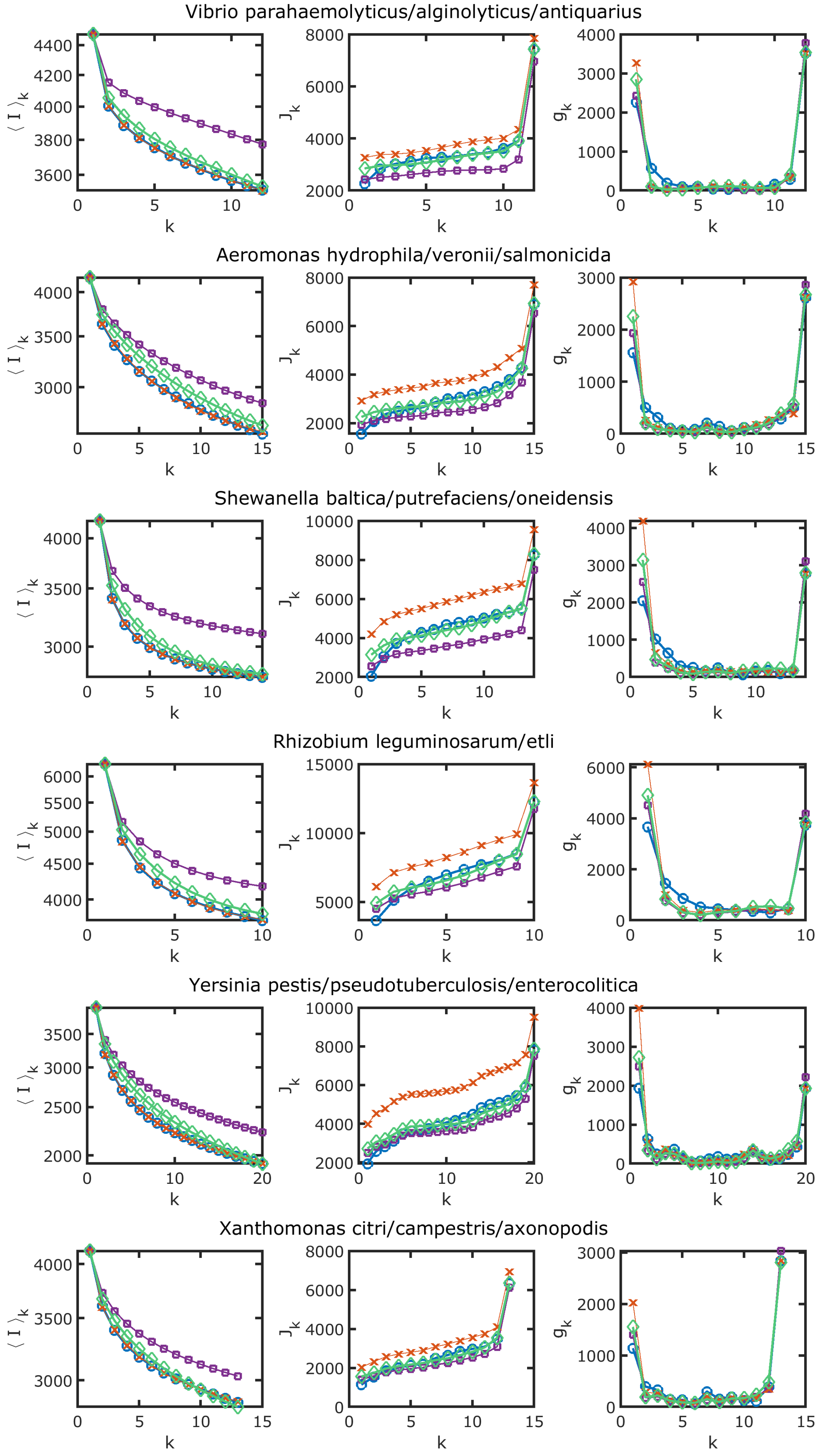

FIG. S10: Same as Fig. S7.

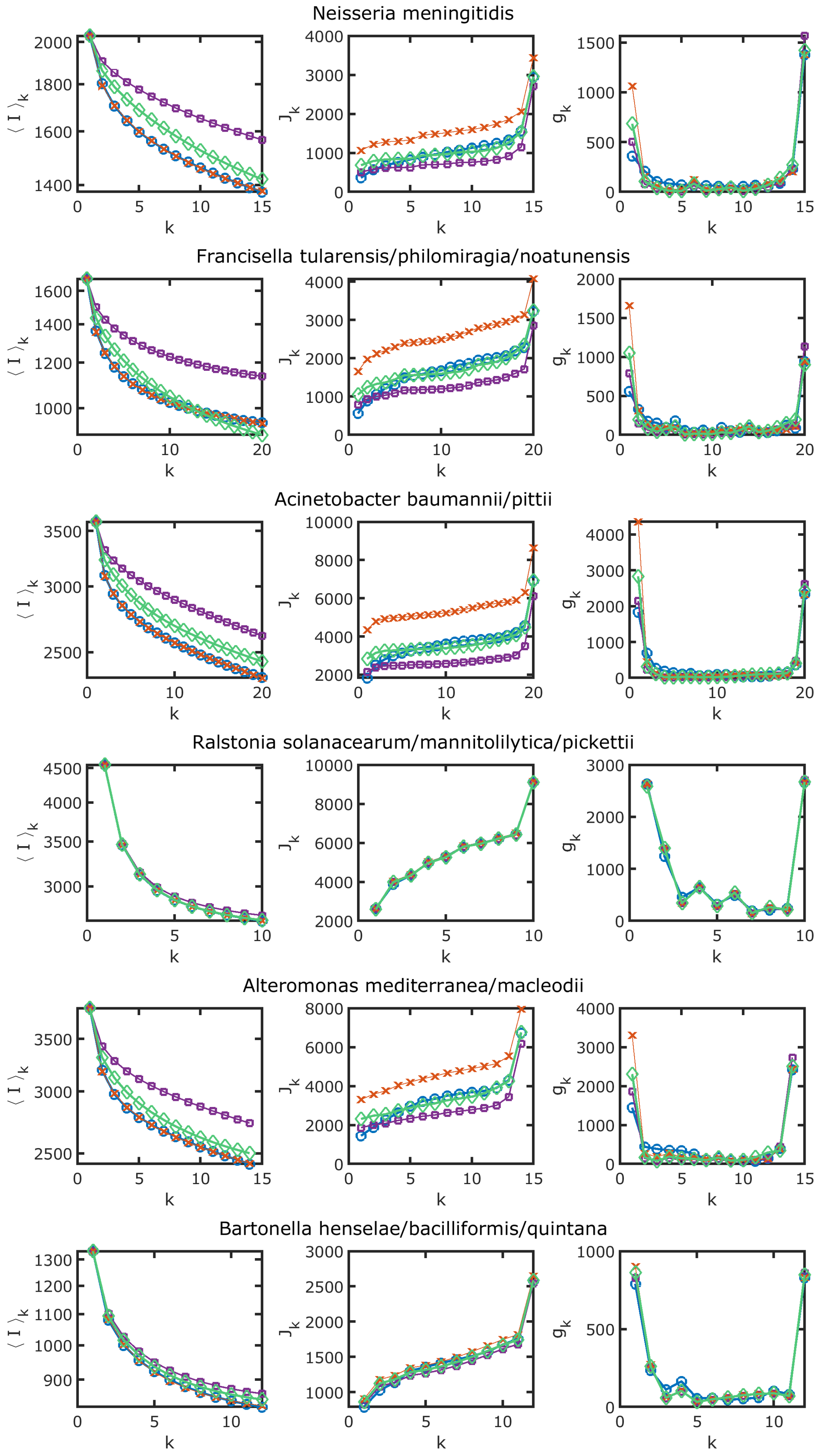

FIG. S11: Same as Fig. S7.

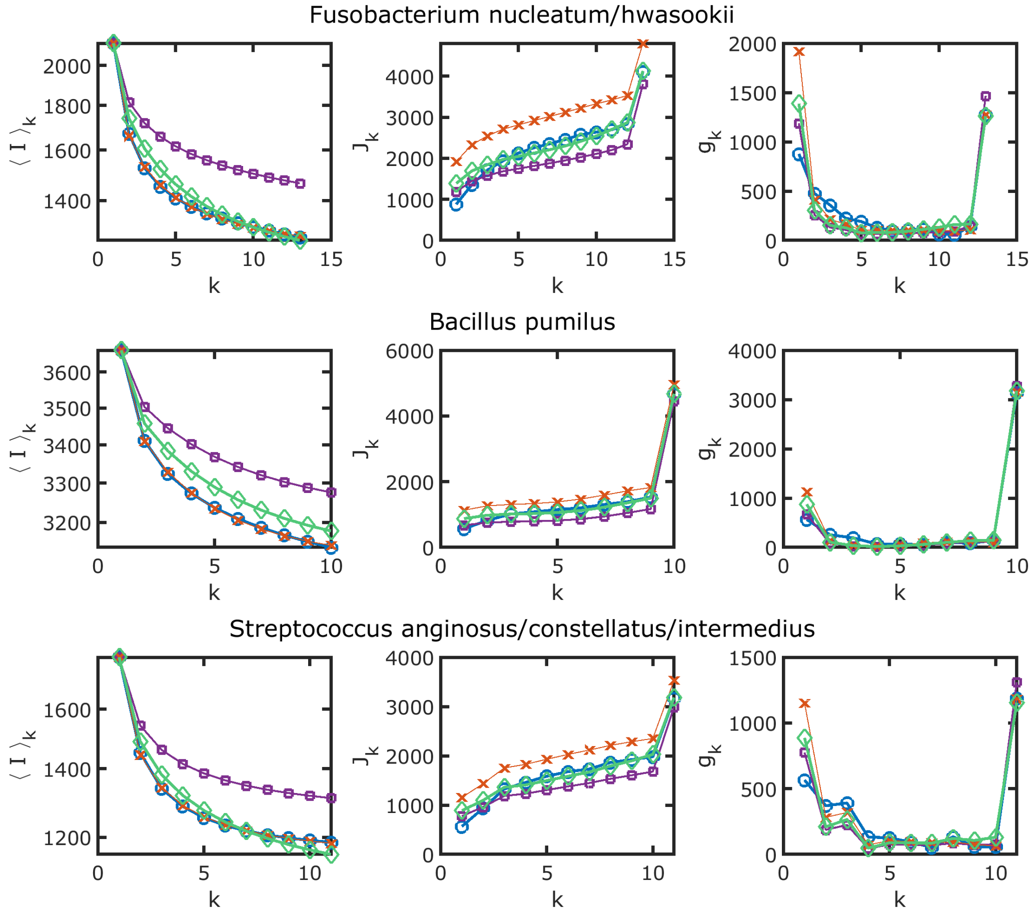

FIG. S12: Same as Fig. S7.

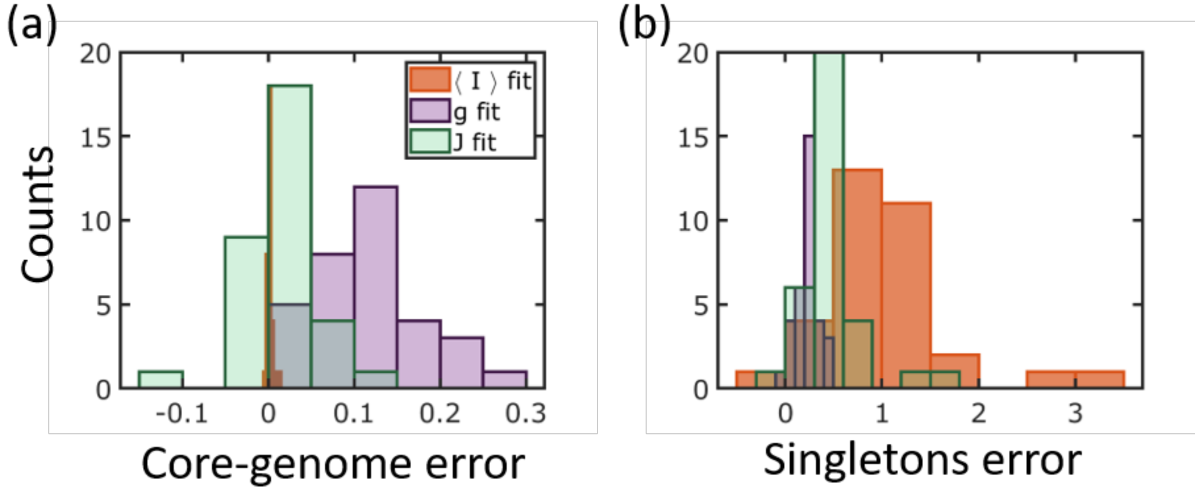

FIG. S13: Comparison of the statistics of the IGP-CGS model fit to the 33 genomic clusters, when fitted to mean intersections $\langle I \rangle_k$, gene commonality g_n, and gene cumulative commonality J_k. **a)** Histogram for the error in core-genome sizes g_N of model fit. The error is calculated as $\left(g_N^{model} - g_N^{data}\right)/g_N^{data}$. **b)** Histogram of the error in model prediction for the number of singletons, as computed from model mean genomes intersections using Eq. 3. The error is calculated as in panel **a**.

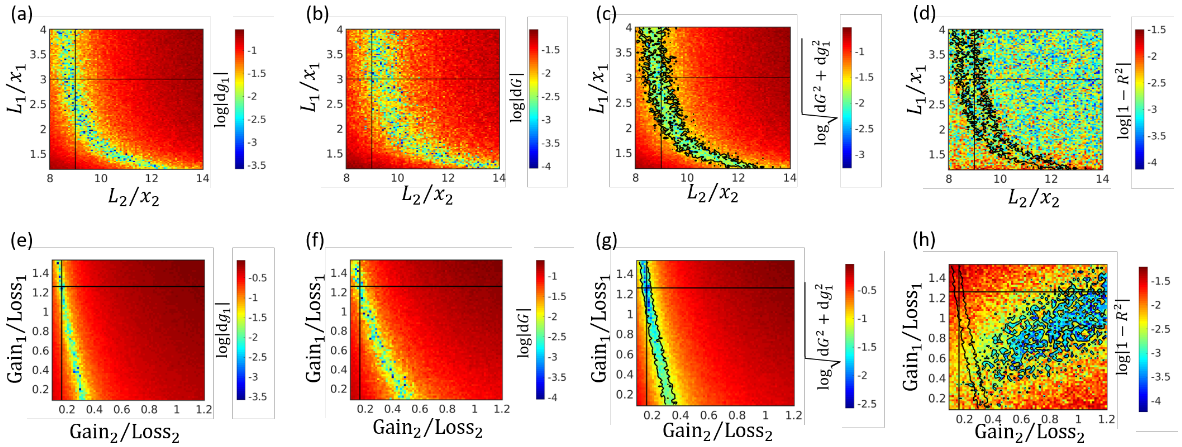

FIG. S14: The similarity of simulated datasets to the genomic data of ATGC001. Simulations for different pool sizes under the FGP-CGS assumption are shown in panels **a-d**. Simulations for different gain to loss ratios under the IGP-VGS assumption are shown in panels **e-h**. The similarity between the simulated data is quantified by the error in the number of singletons (panels a and e), the error in the pangenome size (panels **b** and **f**), a combined measure of the number of singletons and the pangenome size (panels **c** and **g**), and the goodness of fit for the mean intersections (panels **d** and **h**). the error dX is calculated as $(X_{model} - X_{data})/X_{data}$. Contour lines of the optimal combined measure are indicated in panels **c** and **g**. For comparison, the optimal region in terms of the combined measure is also shown in panels **d** and **h**. The parameters that were used in the simulations that are shown in Fig. 8 of the main text are indicated by a vertical and a horizontal black lines in all panels.
